# RUNX1 Upregulates CENPE to Promote Leukemic Cell Proliferation

**DOI:** 10.3389/fmolb.2021.692880

**Published:** 2021-08-09

**Authors:** Shan Liu, Jianyu Yang, Guohuan Sun, Yawen Zhang, Cong Cheng, Jin Xu, Kuangyu Yen, Ting Lu

**Affiliations:** ^1^ School of Biology and Biological Engineering, Southern China University of Technology, Guangzhou, China; ^2^ State Key Laboratory of Experimental Hematology, National Clinical Research Center for Blood Diseases, Institute of Hematology and Blood Diseases Hospital, Chinese Academy of Medical Sciences and Peking Union Medical College, Tianjin, China; ^3^ Department of Developmental Biology, School of Basic Medical Sciences, Southern Medical University, Guangzhou, China; ^4^ Department of Cell Biology, Tianjin Medical University, Tianjin, China; ^5^ Division of Cell, Developmental and Integrative, School of Medicine, Southern China University of Technology, Guangzhou, China

**Keywords:** Runx1, leukemia cell, cell cycle, apoptosis, differentiation potential

## Abstract

RUNX1 is a Runt family transcription factor that plays a critical role in normal hematopoiesis, including the differentiation and proliferation of hematopoietic cells. RUNX1 mutations, including chromosomal translocations, cause abnormal cell differentiation, but the mutation alone is not sufficient to cause leukemia. In MLL-fusion-induced leukemia, dysregulated wild-type RUNX1 can promote leukemia survival. Nevertheless, the underlying mechanisms of dysregulated wild-type RUNX1 in leukemia development have not been fully elucidated. This study overexpressed and knocked down RUNX1 expression in THP-1 human leukemia cells and CD34^+^ hematopoietic stem/progenitor cells to investigate the biological functions affected by dysregulated RUNX1. Our data indicated RUNX1 facilitated proliferation to promote leukemia cell growth. Furthermore, we demonstrated that RUNX1 knockdown in leukemia cells drastically diminished colony-forming ability. Finally, the RUNX1-knocked down cell depletion phenotype could be rescued by overexpression of CENPE, a cell proliferation gene and a RUNX1 direct target gene. Our results indicate a possible mechanism involving the RUNX1-CENPE axis on promoting leukemic cell growth.

## Introduction

Transcription factor RUNX1, also known as AML1 or CBFA-2, plays a pivotal role in normal hematopoiesis. In normal embryonic cells, RUNX1 knockout embryos could not promote vascular endothelial cells to differentiate into definitive hematopoietic cells and died at embryonic day (E) 12.5 (Okuda et al., 1996). In adult hematopoiesis, RUNX1 is constitutively expressed in bone marrow hematopoietic stem and progenitor cells, which can further differentiate into all hematopoietic lineages cells, with the exception of erythrocytes (Wang et al., 1996; North et al., 2004). Moreover, conditional RUNX1 knockout in mice bone marrow displayed hematopoietic differentiation failure. RUNX1 was essential for cell cycle progression and proliferation in murine erythroid progenitor cells, and knockdown of RUNX1 caused attenuated proliferation in bone marrow-derived mesenchymal stem cells (Keita et al., 2013; Kim et al., 2014). During the early stages of erythropoiesis, RUNX1 promotes murine erythroid progenitor proliferation by preventing Pu.1 downregulation (Willcockson et al., 2019). In addition, the loss of RUNX1 in primary murine or human myeloid progenitors markedly impaired murine marrow cell proliferation (J D’Costa1 et al., 2005). Altogether these findings illustrated the indispensable role of RUNX1 in hematopoiesis, especially in differentiation and proliferation.

RUNX1 mutations caused by chromosomal rearrangements, fusion proteins, or point mutations are commonly observed in leukemia. RUNX1 translocations usually form fusion proteins responsible for new functions that are nonexistent in normal RUNX1 protein. For example, ETO in the t (8; 21) chromosomal aberration forms a fusion protein with RUNX1 and inhibits myeloid differentiation in bone marrow cells (Yuan et al., 2001). However, current studies have found that RUNX1-ETO can also cause abnormal cell differentiation, but the fusion protein itself is insufficient to develop into acute myeloid leukemia (AML) unless there are additional mutations (Nishida et al., 2006; Becker et al., 2008). These results were consistent with the predominant hypothesis on leukemogenesis, known as the “two-hit” model, which suggests that leukemia development requires the existence of a combination of two gene alterations (Knudson, 1971; Conway O’Brien and Steven, 2014). “One hit” would result in uncontrolled cellular proliferation (Shih1 et al., 2012), while “the second hit” is associated with differentiation failure, including key transcription factors RUNX1 (Silva et al., 2003).

Other than RUNX1 mutations, there has been increasing recognition of the importance of dysregulated wild-type RUNX1 in leukemogenesis in recent years. Many leukemic diseases, especially AML, display an abnormally elevated expression of wild-type RUNX1 (Li et al., 2019; Sun et al., 2019). Loss of wild-type RUNX1 significantly reduces cell proliferation in CBFB-MYH11 fusion-induced leukemia cells (Hyde et al., 2015), E2A-PBX1-induced ALL (Pi et al., 2020), and MLL-fusion-induced AML (Milne et al., 2002). Inhibition of RUNX1 using the chemical inhibitor Ro5-3335 (Cunningham et al., 2012) in MLL-AF9 leukemia cells causes cell number depletion or cell cycle arrest and suppressed leukemia development (Goyama et al., 2013). These findings suggest that dysregulated wild-type RUNX1 may promote MLL leukemia cell survival (Cunningham et al., 2012; Goyama et al., 2013; Ke, 2016). Besides the causative RUNX1 mutation in leukemia diseases, dysregulated wild-type RUNX1 also contributes to leukemia development. However, the underlying mechanism of dysregulated wild-type RUNX1 in leukemogenesis remains unclear.

Herein, we identified RUNX1-targeted biological functions in AML by examining publicly available transcriptome profiles and RUNX1 ChIP-seq datasets of THP-1 and CD34^+^ cells. We validated these RUNX1-targeted biological functions by overexpressing and knocking down RUNX1 expression in THP-1 and CD34^+^ cells. We identified CENPE, also known as Centrosome-associated protein E, as the direct target of RUNX1. Overexpressing CENPE can rescue the cell number depletion phenotype caused by RUNX1 knockdown in the THP-1 cell line. Our results suggested that RUNX1 could induce leukemia cell growth by promoting cell proliferation, which was regulated in part by the RUNX1-CENPE axis. Our results pave the way for further exploration of the molecular mechanisms associated with dysregulated RUNX1 in leukemia.

## Materials and Methods

### Ethics Statement

Ethical approval for this study was obtained from the Ethics Committee of Blood Diseases Hospital, Chinese Academy of Medical Sciences, and required the anonymization of sample data to ensure patients could not be identified. The authors had no access to any patient information. According to the regulations of the institutional ethics review boards from the Institute of Hematology and Blood Diseases Hospital, Chinese Academy of Medical Sciences and Peking Union Medical College, informed consent was signed by all patients*.*

### Standard Biosecurity and Institutional Safety Procedures

Our experimental operations and environment comply with standard biosecurity and institutional safety procedures.

### Cell Lines

The 293T and THP-1 cell lines were obtained from the State Key Laboratory of Experimental Hematology, Beijing, China. Both cell lines were confirmed to be mycoplasma-free.

### Human Samples

CD34^+^ cells were obtained from umbilical cord blood using a CD34^+^ MicroBead Isolation Kit (Miltenyi) using samples from the Tianjin Central Hospital of Gynecology and Obstetrics.

### Cell Culture

293T cells were maintained in DMEM medium with 10% FBS (Gibco). THP-1 cells were cultured in RPMI 1640 Medium (Gibco) with 10% FBS. CD34^+^ cells were cultured in SFEM (Serum-Free Expansion Medium) medium with 100 ng/ml SCF (stem cell factor), 50 ng/ml Flt3 (Fms Related Receptor Tyrosine Kinase 3), 50 ng/ml TPO (Thrombopoietin) (all from PeproTech), and 1% penicillin/streptomycin (Gibco). All cell expansions were performed using ultra-low attachment surface tissue culture plates (Corning).

### Plasmids and Virus Production

The pCDH-CMV-EFi-coGFP vector was generously provided by the Jianxiang Wang lab. Human RUNX1 cDNA was inserted into the vector to form the pCDH-CMV-hgRUNX1-EFi-CoGFP construct. The shRNA plasmids were purchased from the Shanghai Genechem company and encoded for RUNX1-RNAi (38562-1/2/3) and shControl (CON053). All overexpression and shRNA plasmids were transfected into 293T cells and downstream effects were confirmed by qPCR and western blotting. For lentiviral production, the RUNX1 target plasmid was transfected together with pSPAX2 and pMD2G into 293T cells using Lipo3000 and the supernatant was collected after 24 and 48 h and filtered using a 0.45 μm Amicon filter. The viral vectors were concentrated by ultracentrifugation.

### RNA Extraction and Quantitative Real-Time Polymerase Chain Reaction

Total RNA was isolated from 1 × 10^7^ cells using a TRizol reagent (Roche). The first-strand RNA was synthesized from 2 μg of total RNA using dT primer. Furthermore, cDNA was synthesized with the iScript™ Advanced cDNA Synthesis Kit for subsequent quantitative real-time polymerase chain reaction (qRT-PCR) (Promega), which was performed on a Q3 real-time PCR system (Applied Biosystems). The primers used in this study are listed in Supplementary Table S2. All reactions were normalized against GAPDH levels. Statistical significance was determined with a significance threshold of *p* < 0.05.

### Western Blotting

Cell extracts were prepared using Cell lysis buffer (Cell Signaling, #9803S) supplemented with protease inhibitors. Protein lysates were cleared by centrifuging at 12,000 rpm for 30 min at 4°C and boiled with SDS-loading buffer at 95°C, for 10 min. Thereafter, the membrane was probed using antibodies against RUNX1 (Cell Signaling, 2527S, 1:500).

### Flow Cytometry

Flow cytometry (FC) was conducted using the MACSQuant® Analyzer 10 (Miltenyi Biotec) and the following antibodies: CD34-FITC (Stemcell), ki67-PE, Annexin V-APC, and 7-AAD (all bought from Biolegend).

### Apoptosis and Proliferation Assays

For apoptosis detection, cells were stained with CD34-FITC (BD Pharmingen), along with Annexin V-PE (BD Pharmingen), and analyzed by flow cytometry. To assess cell proliferation, cells were incubated with cell proliferation antigen Ki-67. According to the manufacturer’s protocol, cells were then fixed and permeabilized with 70% ethanol and stained with Ki67-APC (Miltenyi Biotech); the collected samples were further analyzed by FC. Cell cycle analysis of total hematopoietic progenitors was carried out using the FITC BrdU flow kit (BD Pharmingen), following the manufacturer’s recommendations.

### Colony-Forming Assay

For both the overexpression and shRNA conditions, the colony-forming potential of THP-1 and CD34^+^ cells was assessed by combining the cells of interest (1,000 cells) with 0.5 ml serum-containing MethoCult H4034 Optimum (Stem Cell Technologies). Cell suspensions were then transferred to 24 well-plates dishes and cultured for 12–14 days at 37°C. Colonies were scored according to cellular morphology, and CFU numbers were normalized to the number of empty vector THP-1/CD34^+^ plated cells.

### Data Quality Control

Fastqc was used to assess the quality of all datasets. The per base sequence quality and sequencing depth of each sample were summarized by MultiQC (Ewels et al., 2016). The fraction of reads in peaks (FRiP) of each ChIP-seq sample were computed by counting the number of reads intersected with peaks, then divided by total number of reads.

### RNA-Seq Analysis

Trimmomatic software was used to trim the adapter sequences and low-quality bases, and the remaining reads were mapped to the reference genome (human: hg38) using STAR with the “--quantMode GeneCounts” option enabled. The gene count table for each experiment was further combined and input to DESeq2 for differential gene analysis. Genes with *P*adj < 0.05 and abs (log2 FoldChange) > 1 were selected as the differentially expressed genes for downstream analysis. Human HSPC RNA-seq data were downloaded from GEO (GSM2797289, GSM2797288), while THP-1 RNA-seq data were obtained from GEO (GSM2599707, GSM2599708).

### ChIP-Seq Analysis

Trimmed reads were mapped to the reference genome (human: hg38) by Bowtie2 with the default option. The mapped reads with MAPQ > 10 were retained. Peak calling was performed by MACS2 using the shifting model and *q*-value 0.05 as cutoff, and the resulting narrow peak file was used for the downstream analysis. Human HSPC RUNX1 ChIP-seq data were from GEO (GSM1816091, GSM1816092) and THP-1 cell RUNX1 ChIP-seq data were from GEO (GSM2108052).

### Differential Genes Regulated by RUNX1

The differentially expressed genes regulated by RUNX1 were obtained by checking whether at least one RUNX1 peak was located within the ± 3,000 bp region or around the transcription start sites. THP1-specific RUNX1 target genes were defined as the set of differentially expressed genes with at least one RUNX1 peak located within ± 3k bp region around the transcription start site only in THP1 cells.

### Gene Ontology Term Analysis

Gene Ontology (GO) term enrichment was performed on the up/down-differentially expressed genes regulated by RUNX1 with gProfiler. The “ordered_query” option allowed to obtain rank information ordered by *p*adj from the DESeq2 package.

### Statistical Analysis

Statistical analysis was performed using Prism (GraphPad, San Diego, CA, United States) using the student ANOVA. Data were reported as the median ± SD of at least three independent experiments. Results with a *p-*value < 0.05 were considered statistically significant.

## Results

### RUNX1 in Leukemia Cells Regulated Cell Cycle-Associated Biological Processes

Aberrantly expressed wild-type RUNX1 has been observed in several leukemias, although the mechanisms involved in promoting leukemia development are still largely unknown. To investigate the role of RUNX1, we selected the THP-1 cell line. THP-1 cells exhibit aberrantly high RUNX1 expression and the addition of a RUNX1 inhibitor can greatly reduce leukemia cell number (Goyama et al., 2013). Furthermore, THP-1 cells are acute monocytic leukemia cells with hindered differentiation (Herbert and Heinzelmann, 2016). They are at a less mature stage (Nonnemacher et al., 2017) and display a relatively high stemness (CD34^+^ phenotype) (Nasser et al., 2021). For comparison, we used CD34^+^ hematopoietic cells.

**FIGURE 1:**
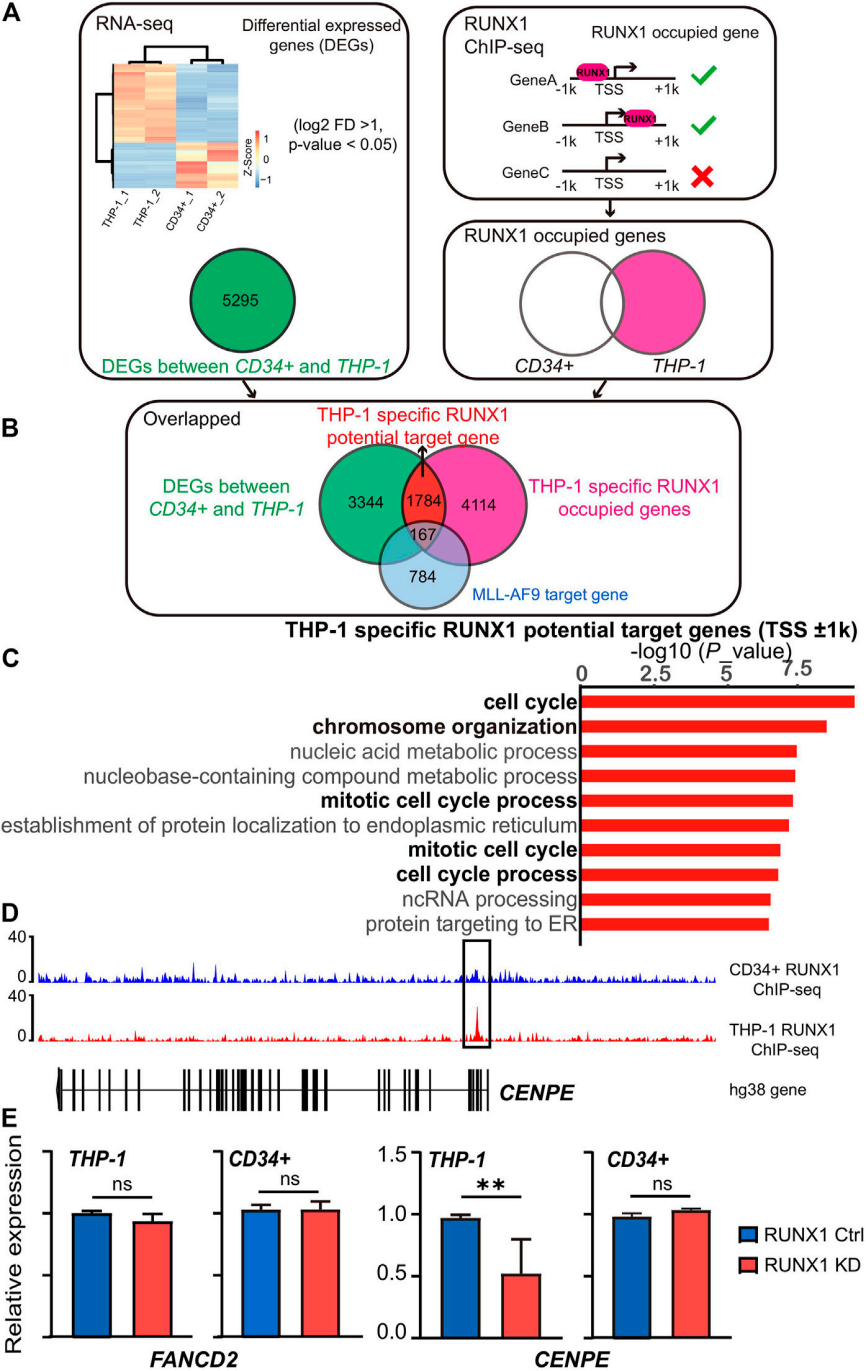
RUNX1 in leukemia cells regulates cell cycle-associated biological process. **(A)** Workflow for choosing differential expression genes and the RUNX1 occupied genes. Heat map showed the RNA-seq differentially expressed genes (DEGs) (FDR *p* < 0.1 and >2 -fold change) between THP-1 cells and CD34^+^ cells. RUNX1 occupied genes was defined as genes contained RUNX1 binding around ±1 kb relative to TSS. **(B)** Workflow for choosing THP-1 specific RUNX1 potential target genes. For THP-1 specific RUNX1 potential target genes, we overlapped DEGs with THP-1 specific RUNX1 occupied genes. **(C)** Gene Ontology (GO) analysis of 1784 differentially expressed genes that contain RUNX1 binding site but without MLL-AF9 occupied in promoter regions (±1 kb relative to TSS) for THP-1 cells. Black font indicates the biological pathways that are related to cell growth; gray font indicates other biological pathways. **(D)** RUNX1 peaks located in the proximal vicinity of TSSs of CENPE. **(E)** qPCR confirmed FANCD2 and CENPE mRNA expression of THP-1 cells and CD34^+^ cells with or without RUNX1 shRNA. (one-way ANOVA, *p < 0.05, **p < 0.01,***p < 0.001; error bars, median ± SD).

To understand the role that dysregulated wild-type RUNX1 plays to promote leukemia development, we needed to identify potential THP-1-specific RUNX1 target genes. To achieve this, we first identified genes specifically affected in THP-1, but not in CD34^+^ cells. We identified all differentially expressed genes (DEGs) between THP-1 and CD34^+^ cells using publicly available transcriptome profiles (GSM2797289 and GSM2599707, respectively) (Douglas et al., 2017; Smith et al., 2020). Quality control showed that all samples had sufficient sequencing depth and high per base quality (Supplementary Figure S1). Overall, 5295 DEGs were identified (log2 fold change > 1, adjusted *p*-value < 0.05) (Figure 1A). RUNX1 was among these DEGs and, as expected, was upregulated in THP-1 cells, and this upregulation was further confirmed using qRT-PCR (Supplementary Figure S2A). We then applied GO enrichment analysis on these DEGs to identify which biological pathways were dysregulated in THP-1 cells. The top 10 dysregulated biological pathways in THP-1 cells included cell division, cell cycle, and DNA replication (Supplementary Figure S2B). It is worth noting that, in these top 10 dysregulated cell growth-related pathways, almost all the affected genes are upregulated, only very few were down-regulated (Supplementary Figure S2C). These results were consistent with the notion that THP-1 cells had a more active cell growth phenotype than CD34^+^ cells (Nonnemacher et al., 2017). Since THP-1 cells are monocytic leukemia, to clarify if our finding is caused by different cell types or direct evidence of Runx1 function in leukemia, we further compared THP-1 cells against monocyte. We downloaded a published monocyte transcriptome profile (GSE122682) (Rosa et al., 2021) and compared it with THP-1 cells. We calculated all differentially expressed genes (DEGs) between THP-1 and monocyte cells. Totally, 6477 DEGs were identified (log2 fold change > 1, adjusted *p*-value < 0.05). RUNX1 was among these DEGs and, as expected, shown upregulated in THP-1 cells. We then applied GO enrichment analysis on these DEGs to identify dysregulated biological pathways in THP-1 cells. The top 10 dysregulated biological pathways in THP-1 cells include cell cycle process, mitotic cell cycle, and chromosome segregation (Supplementary Figure S2D). These results are consistent with our previous comparison between THP-1 cells and CD34^+^ cells (Supplementary Figure S1B).

We then identified THP-1 specific RUNX1 potential target genes. We hypothesized that potential RUNX1 target genes were genes that contained RUNX1 binding sites in their promoter regions and its expression changed on RUNX1 binding. Using this assumption, we defined potential THP-1-specific RUNX1 target genes as DEGs whose promoters contained RUNX1 binding sites in THP-1 cells, but not in CD34^+^ cells. We took advantage of available RUNX1 ChIP-seq datasets generated from THP-1 cells (GSM2108052) and CD34^+^ HSPCs (GSM1816092) (Huang et al., 2016; Prange et al., 2017). We mapped ChIP-seq reads onto the human genome (hg38) using bowtie2 before peak calling by MACS2 to identify the binding sites for RUNX1. Among the 5295 DEGs (Figure 1A), 1951 DEGs exhibited RUNX1 binding around ±1 kb relative to its TSS in THP-1 cells, whereas 180 DEGs did not present RUNX1 binding (Figure 1B). To further clarify if these DEGs are the genuinely independent mechanism by RUNX1 and not a direct function of the MLL-AF9 fusion protein, we overlapped these genes with a defined THP-1 MLL-AF9 target gene cohort (Prange et al., 2017). 167 out of the 1951 DEGs are also considered as MLL-AF9 target genes (Supplementary Figure S3A). To avoid complications from MLL-AF9 fusion protein, the rest of 1784 DEGs are regarded as potential THP-1-specific RUNX1 target genes. We further applied GO enrichment analysis to this gene set; and again, cell growth-related biological pathways dominated the top 10 enriched biological pathways (Figure 1C). We focused on the top three potential THP-1-specific RUNX1 target genes involved in cell growth-related pathways, including CENPE, CLSPN, and XPOI. We observed RUNX1 binding in the proximal vicinity of TSSs of these three genes in THP-1 cells, but no RUNX1 binding was observed in the same region in CD34^+^ cells (Figure 1D; Supplementary Figure S3B).

To further validate whether the aberrantly expressed wild-type RUNX1 in THP-1 cells contributed to the regulation of cell growth-related genes, we examined the changes in gene expression in THP-1 cells when exposed to RUNX1 shRNA. To confirm this regulation was THP-1-specific, CD34^+^ cells that underwent the same treatment were used as control. The Top 10 potential THP-1-specific cell growth-related genes targeted by RUNX1 were chosen for qRT-PCR validation (Supplementary Figure S4). FANCD2 and ANLN, whose expression was upregulated in THP-1 but contained no RUNX1 binding sites on their promoter regions, were chosen as control. As expected, when THP-1 were treated with RUNX1 shRNA, FANCD2 and ANLN showed no change in expression. Eight of the top 10 potential THP-1-specific RUNX1 target cell growth-related genes, apart from XPOI and ZMPSTE24, showed a reduced expression in THP-1 cells whereas the same genes in CD34^+^ cells showed modest to no change (Figure 1E; Supplementary Figure S4). These results indicated that the dysregulated wild-type RUNX1 regulated cell growth-related genes in THP-1 cells, but not in CD34^+^ cells.

In summary, aberrantly expressed wild-type RUNX1 in THP-1 cells mainly upregulated the expression of genes involved in the cell cycle and DNA replication, which may contribute to the more the active growth phenotype seen in THP-1 cells.

### RUNX1 Affects Leukemia Cell Growth and Differentiation

**FIGURE 2:**
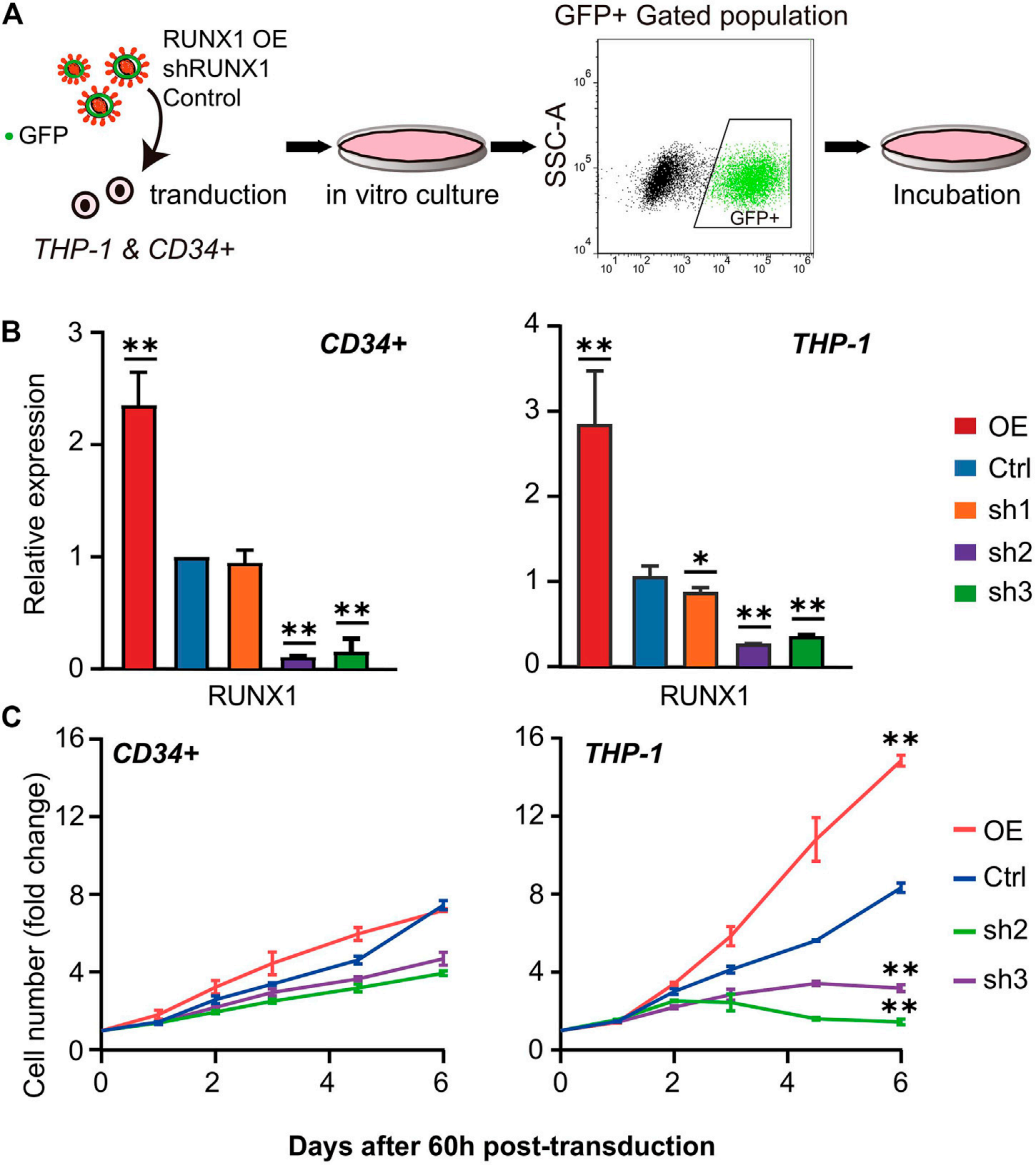
Cell number phenotype caused by RUNX1 overexpression (OE) and knockdown (KD). **(A)** Scheme of lentivirus transduction experiments. RUNX1 OE and KD virus were transducted into THP-1 and CD34^+^ cells. After FC sorting, GFP + cells were transferred into fresh medium and culture *in vitro* for further experiment. **(B)** qRT-PCR confirmed the relative RUNX1 mRNA expression of THP-1 and CD34^+^ cells with empty vector, RUNX1 and RUNX1 shRNA *in vitro*. ALL groups were compared with Ctrl. All reactions were normalized against GAPDH. **(C)** THP-1 and CD34^+^ cells were transfected with the RUNX1 and RUNX1 shRNA virus, followed by efficiency check at 60 h post-transduction before growth curves were monitored daily. The cell number was normalized to Day 0 before plotting. Blue trace is the empty vector control. Red, green, and purple traces are cells transfected with individual RUNX1, RUNX1 sh1 and sh2, respectively. (one-way ANOVA, *p < 0.05, **p < 0.01,***p < 0.001; error bars, median ± SD).

Our analysis suggested the biological processes regulated by the potential THP-1-specific RUNX1 target genes were mainly related to the cell cycle and DNA replication. To further verify whether RUNX1 affects these processes, we overexpressed (OE) and knocked down (KD) RUNX1 expression in THP-1 and CD34^+^ cells. As shown in Figure 2A, we transduced THP-1 and CD34^+^ cells with green fluorescent protein (GFP) containing lentivirus that harbored RUNX1 shRNA or RUNX1 OE. A no-targeting shRNA as a negative control was also transduced in both cell models. We monitored virus concentration, cell doubling time, and transfection time point to optimize transduction efficiency (Supplementary Figures S5A–C). As a result, transduced THP-1 and CD34^+^ cells were cultured *in vitro* for 60 h before FC analysis to identify cells that underwent successful transduction. The effects of RUNX1 KD or OE in THP-1 and CD34^+^ cells were verified by qRT-PCR and western blotting (Figure 2B; Supplementary Figure S6A). In the OE RUNX1 groups, RUNX1 expression was significantly increased in THP-1 and CD34^+^ cells (Figure 2B). For KD RUNX1 groups, one of the three RUNX1 shRNA (sh1) did not alter RUNX1 expression significantly in CD34^+^ cells and only modestly affected THP-1 cells. The other two RUNX1 shRNAs (sh2 and sh3) used showed significantly decreased RUNX1 expression. Therefore, we used the latter RUNX1 shRNAs (sh2 and sh3) in subsequent experiments.

We first seeded equal amounts of transduced cells and recorded the growth curve for 7 days. We observed increased cell numbers in RUNX1 OE transduced cells in both THP-1 and CD34^+^ cells (Figure 2C). This increase in cell numbers was more significant in THP-1 cells (2-fold increase in cell number) than in CD34^+^ cells. In contrast, the cell numbers in RUNX1 KD transduced cells were reduced (Figure 2C). Specifically, this cell number reduction was much more prominent in THP-1 cells; an average eight-fold decrease was observed in THP1 cells following RUNX1 knockdown, whereas an average 1.5-fold decrease was observed in CD34^+^ cells transduced with RUNX shRNA.

**FIGURE 3:**
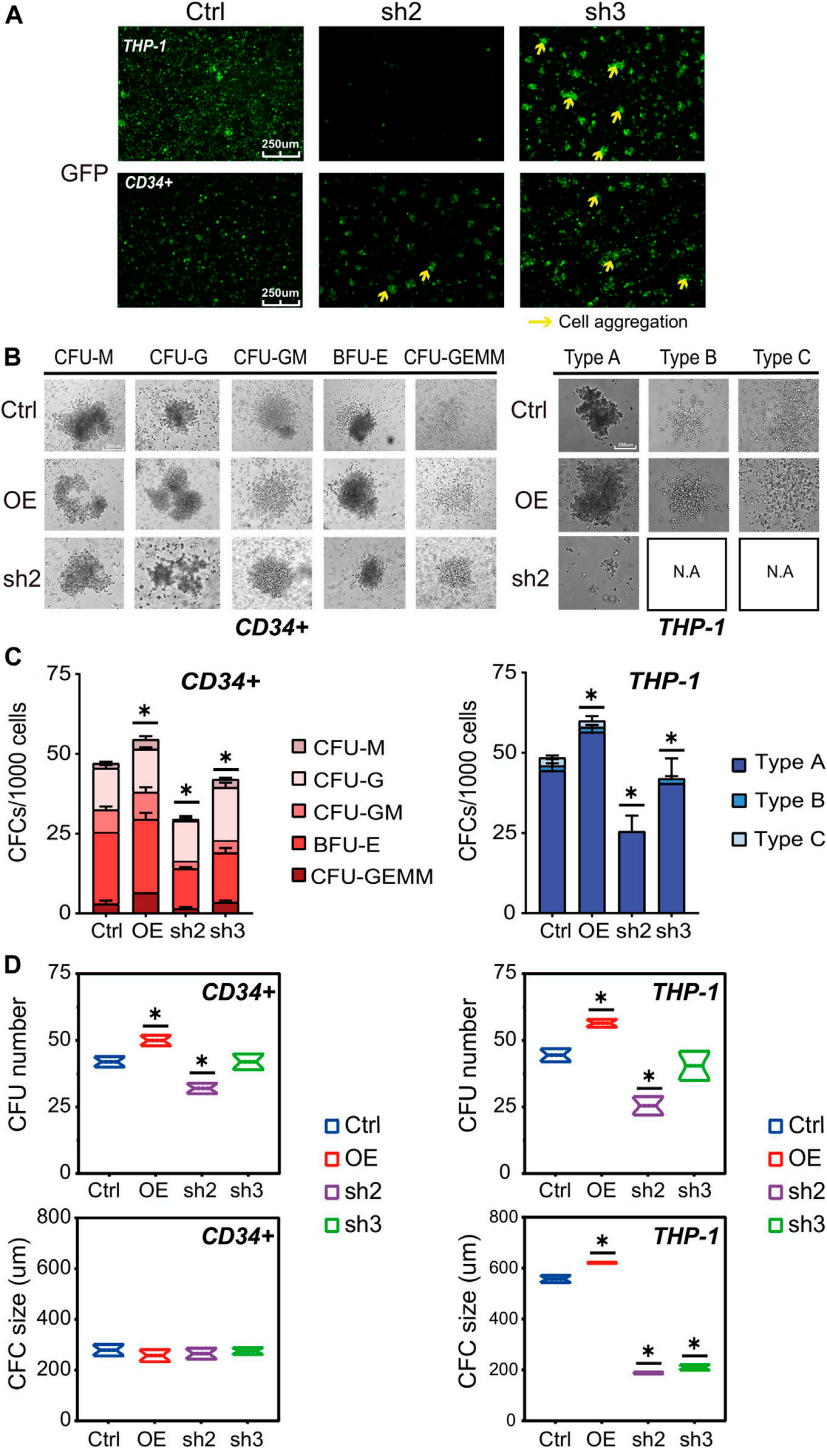
RUNX1 knockdown may affect leukemia cell differentiation ability. **(A)** Cell morphology change and cell aggregation in cell culture at day 5. **(B)** Colony formation of human THP-1 cells and CD34^+^ cells after transduction of empty vector, RUNX1 and shRUNX1. The colony morphology could be observed at **(B)** and the whole colony number and subtype calculated at **(C)**, colony number (upper) and colony size (lower) were respectively analyzed at **(D)**. (one-way ANOVA, *p < 0.05, **p < 0.01,***p < 0.001; error bars, median ± SD).

Besides the changes in cell numbers (Figure 2), we also evaluated the cell aggregation phenotype of THP-1 and CD34^+^ cells with RUNX1 KD (Figure 3A). This aggregation phenotype was much more prevalent in THP-1 cells. Previous studies have shown that the cell aggregation phenotype might reflect its differentiation potential (HUSS, 2000; Cheng et al., 2000; Toyoda et al., 2015). RUNX1 has long been seen as a master regulator that facilitates hematopoietic differentiation (Ichikawa et al., 2013). Thus, we speculated that RUNX1 could also have an impact on THP-1/leukemia cell differentiation potential.

The differentiation potential of RUNX1 in THP-1 and CD34^+^ cells was evaluated using colony-forming assays (Wognum et al., 2013). A total of 1,000 cells were seeded in a 96-well plate and incubated for 15 days before microscopic inspection. As expected for CD34^+^ cells (Chotinantakul et al., 2013), we observed five colony subtypes in CD34^+^ cells transfected with control no-target lentivirus (Figure 3B, left panel). Regardless of RUNX1 OE or KD treatment, CD34^+^ cells also displayed the same five colony subtypes. Conversely, RUNX1 OE in CD34^+^ cells increased the total number of colonies formed, whereas RUNX1 shRNA led to a reduced number of colonies (Figures 3C,D). No significant differences in colony size were observed among CD34^+^ groups regardless of treatment (Figure 3D). Moreover, with regard to lineage differentiation, the ratio of CFU-M and CFU-GEMM in RUNX1 OE groups showed the greatest increase among all colony subtypes (Figures 3C,D). These results indicated that RUNX1 expression influenced the differentiation potential of CD34^+^ cells mainly by inducing myeloid differentiation.

For THP-1 cells, we observed three colony subtypes in THP-1 cells transduced with no-targeting lentivirus (Figure 3B; Supplementary Figure S9), which was similar to what was previously described for MLL-AF9 leukemia cells (Johnson et al., 2003). Notably, type A was the predominant colony subtype, while types B and C were less frequent (Figures 3C,D). Interestingly, we observed all three subtypes in the RUNX1 OE group; the RUNX1 KD group, on the other hand, formed only type A while types B and C almost completely disappeared (Figures 3B–D; Supplementary Figure S9). Similar to what was observed in CD34^+^ cells, RUNX1 OE in THP-1 cells increased the total number of colonies formed, whereas RUNX1 KD showed a reduced number of colonies (Figures 3C,D). Furthermore, the colony size in type A, but not in type B and C, was increased in the RUNX1 OE group (Figure 3D). Conversely, the colony size in type A of the RUNX1 KD group was drastically reduced. To further investigate whether cell differentiation was affected by changes in RUNX1 expression, we evaluated phorbol 12-myristate 13-acetate (PMA)-induced cell differentiation of THP-1 cells following RUNX1 knockdown or overexpression. The results of PMA treatment were also consistent with the results obtained in CFU assays (Supplementary Figure S6B), which suggested that RUNX1 may affect the differentiation potential of THP-1 cells.

Overall, our data suggested that RUNX1 can promote leukemia cell growth and maintain the leukemia cell colony-forming ability.

### RUNX1 Increases Cell Proliferation in Leukemia Cells

**FIGURE 4:**
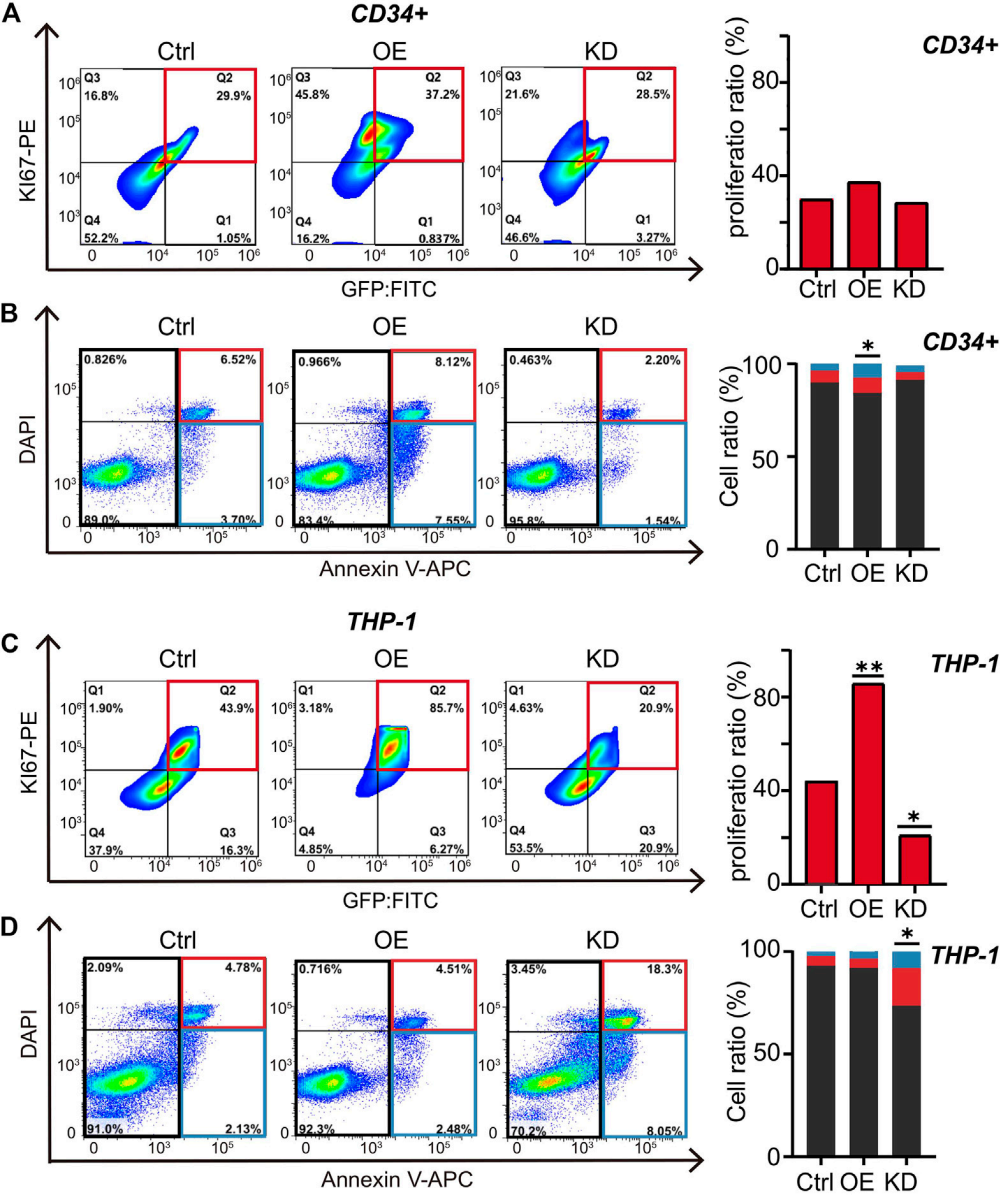
RUNX1 promotes cell proliferation and inhibits cell apoptosis in leukemia cells. **(A)** FC analysis of cell proliferation in THP-1 cells. Flow plots (left) and histograms (right) show the proliferation ratio of THP-1 cells transducted with control, OE (RUNX1), and KD (RUNX1 shRNA). **(B)**. FC analysis of cell apoptosis in THP-1 cells. Flow plots (left) and histograms (right) show the apoptosis (blue and red box) and active cell (black box) ratio. The cell proliferation in **(C)** and apoptosis in **(D)** in CD34^+^ cells were similar described with **(A,B)**. (one-way ANOVA, *p < 0.05, **p < 0.01,***p < 0.001; error bars, median ± SD).

Changes in cell number are mainly influenced by cell proliferation and cell death. To investigate the potential mechanism of dysregulated RUNX1-induced cell number changes, we evaluated cell proliferation and cell apoptosis in both CD34^+^ and THP-1 cells after 5 days of RUNX1 OE and shRNA treatment by FC analysis. A no-targeting shRNA as a negative control was also transfected in both cell lines. We first tested cell proliferation using Ki67-PE, a nuclear protein associated with cellular proliferation, to label cell proliferation (Sun and Kaufman, 2018). For CD34^+^ cells, compared with control cells, the cell proliferation ratio in the RUNX1 OE group was increased, while cell numbers in the RUNX1 KD group were slightly decreased (37.2 and 28.5%, respectively) (Figure 4A). In comparison, changes in RUNX1 induced more substantial effects on cell proliferation in THP-1 cells. The cell proliferation ratio of the RUNX1 OE group in THP-1 cells reached 85.7%, which was nearly a 2-fold increase compared to the 43.9% observed in the control group (Figure 4C). Conversely, in THP-1 cells, the cell proliferation ratio significantly decreased to 20.9% in the RUNX1 KD group.

Although RUNX1 may regulate THP1 cell growth through cell proliferation, whether proliferation is the only process involved is still unknown. Previous studies have shown that RUNX1 also contributes to cell apoptosis (Pulikkan et al., 2018). Thus, we used Annexin V to distinguish apoptotic cells from actively proliferating cells: Annexin V^+^ cells indicated apoptotic cells, while Annexin V^−^ indicated active cells (Figure 4B). In CD34^+^ cells, the ratio of active cells to apoptotic cells from the RUNX1 OE group was significantly higher than in the control group (Figure 4B), which was consistent with previous studies (Jian-Guo et al., 2010). However, knocking down RUNX1 expression in CD34^+^ cells, the apoptotic ratio did not change significantly, which corresponded to the CD34^+^ cell growth curve trend shown in Figure 2C. In contrast, in THP-1 cells, the ratio of active cells to apoptotic cells slightly decreased in the RUNX1 OE group compared to the control group (Figure 4D). Moreover, the apoptotic ratio in the RUNX1 KD group was significantly increased compared to that in the control group. These results suggested that reduced proliferation capacity and the increased apoptosis contributed to the reduced cell proliferation phenotype observed in the RUNX1 KD group of THP-1 cells (Figure 2C).

Overall, our results indicated that RUNX1 promoted leukemia cell growth mainly through up-regulating cell proliferation.

### RUNX1 Regulates CENPE to Promote Leukemia Cell Growth

**FIGURE 5:**
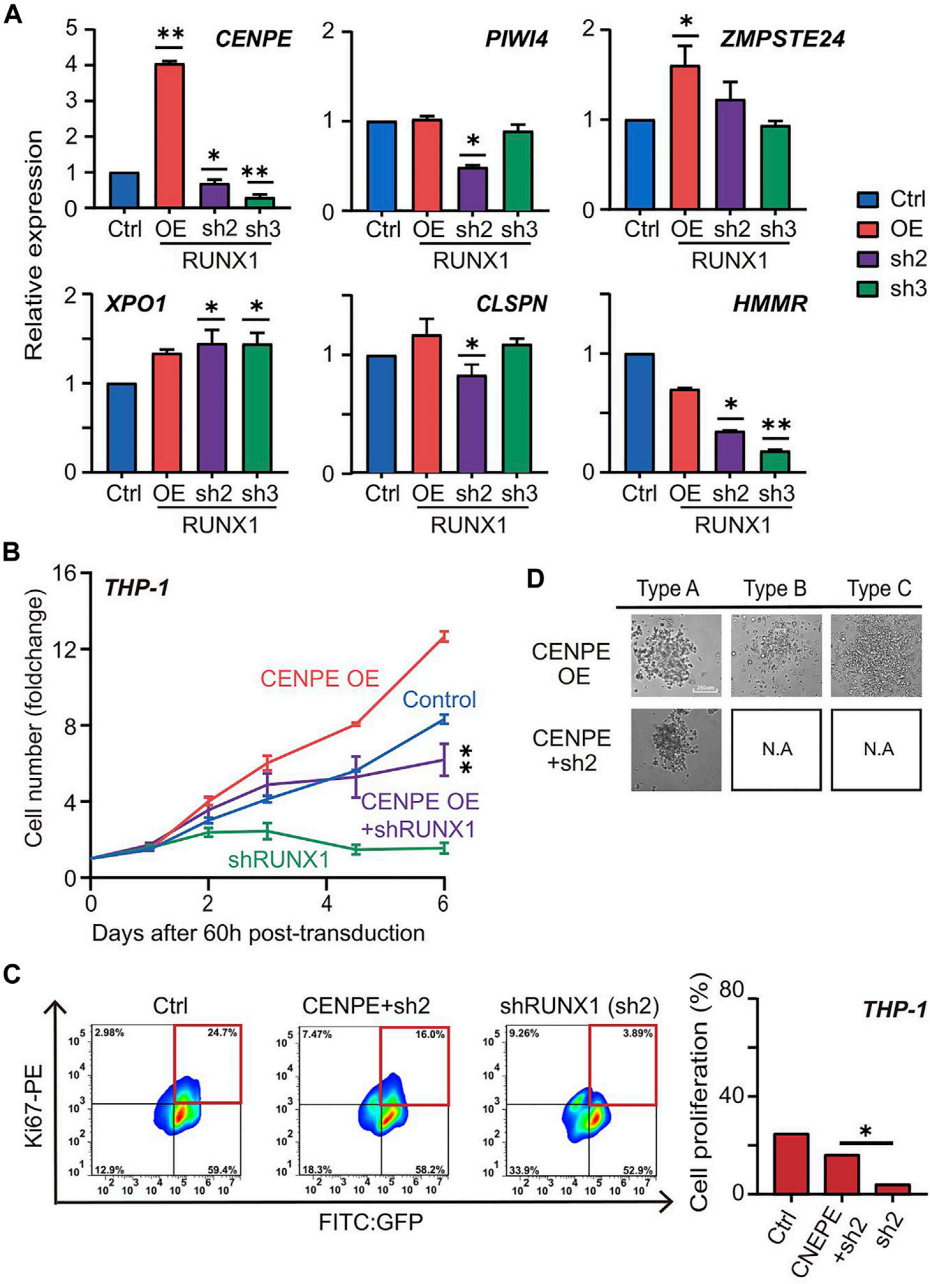
RUNX1 directly regulates CENPE can rescue cell proliferation. **(A)** qRT-PCR confirmed the relative mRNA expression (CENPE, PIWI4, ZMPSTE24, XPO1, CLSPN and HMMR) of THP-1 cells with empty vector, RUNX1 and RUNX1 shRNA *in vitro*. **(B)** Growth curve of THP-1 cells that rescued with CENPE. Growth curve of THP-1 cells that contained different groups. Blue trace is the empty vector control. Orange and green are cells transfected with individual CENPE and shRUNX1, respectively. The purple trace is transfected with CENPE in RUNX1 knockdown of THP-1 cells. **(C)** FC analysis of cell proliferation in THP-1 cells treated with Control, shRUNX1, and shRUNX1+ CENPE. (one-way ANOVA, *p < 0.05, **p < 0.01,***p < 0.001; error bars, median ± SD). **(D)** The colony subtypes of THP-1 cells after transduction of CENPE (labeled as CENPE OE) and CENPE + shRUNX1 (labeled as CENPE + sh2) were observed under the microscope [control (labeled as Ctrl) and shRUNX1 (labeled as sh2) conditions are shown in Figure 3B, but not shown here to avoid redundancy]. Supplementary Figure S9 shows images for all conditions in triplicate.

We further scrutinized the mechanism RUNX1-mediated up-regulation of cell proliferation in THP-1 cells. We focused on the top 10 potential THP-1-specific RUNX1 target genes involved in DNA replication and cell cycle processes (Figure 5A; Supplementary Figure S7A). Since these genes were mainly up-regulated in THP-1 cells, and RUNX1 directly regulates these genes, we expected that the involved candidates would show similar trends in expression trends as that of RUNX1. Using qRT-PCR, THP-1 cells showed increased expression of only CENPE, in the RUNX1 OE group and reduced CENPE expression in the RUNX1 KD group (Figure 5A).

Since CENPE is a centrosome-associated protein that contributes to cell proliferation, we questioned whether RUNX1 regulated leukemia cell growth through CENPE. We first examined the effects of knocking down CENPE in THP-1 cells. No-targeting lentivirus was used as the negative control. Compared with control cells, knocking down CENPE significantly reduced cell numbers of THP-1 cells (Supplementary Figure S7B). Similarly, THP-1 cells overexpressing RUNX1 also produced a reduction in cell number when treated with CENPE shRNA. To further investigate whether CENPE could rescue the phenotype caused by RUNX1 knockdown, we overexpressed CENPE in THP-1 cells with or without concomitant RUNX1 shRNA treatment (Figure 5B). Similarly, a no-targeting lentivirus was used as the negative control. Compared with the control group, we observed a slight, but not significant, increase in cell numbers when overexpressing CENPE in THP-1 cells without RUNX1 shRNA treatment. Nevertheless, overexpressing CENPE in THP-1 cells with RUNX1 knockdown, cell numbers significantly recovered to almost the same levels as those observed in the control group (Figure 5B).

To examine which process CENPE uses to rescue the RUNX1 knockdown-induced THP-1 cell reduction phenotype, we evaluated cell proliferation and cell apoptosis rates in THP-1 cells after 5 days of RUNX1 shRNA and CENPE OE combined treatment using FC analysis (Figure 5C). The no-targeting lentivirus was used as the negative control. Similar to Figure 4C, we observed a reduction in the proliferation ratio in THP-1 cells with RUNX1 shRNA treatment. When rescued by CENPE overexpression, the proliferation ratio significantly recovered to almost the same level as observed in the control group (Figure 5C). However, when we examined cell apoptosis in these same cells, the ratio did not change significantly across the treatment groups (Supplementary Figure S8A). These results indicated that CENPE could rescue RUNX1 knockdown-induced THP-1 cell reduction phenotype through cell proliferation but not *via* reduced apoptosis.

Because RUNX1 shRNA-treated THP-1 cells also showed impaired differentiation potentials, we questioned whether CENPE could further rescue leukemia cell differentiation ability. Using the CFU assay, we seeded 1000 THP-1 cells harboring RUNX1 shRNA and CENPE OE combined treatment in a 96-well plate for 15 days. As shown in Figure 3B, THP-1 cells transfected with no-targeting lentivirus (control) displayed three colony subtypes. Similarly, we observed three colony subtypes in THP-1 cells transfected with overexpressed CENPE (Figure 5D; Supplementary Figure S9). THP-1 cells that knocked down RUNX1 could only differentiate into type A but not type B or C cells (Figure 3B; Supplementary Figure S9). In addition, THP-1 with RUNX1 shRNA treatment displayed a reduced colony size (Figure 3B; Supplementary Figure S9). Interestingly, when rescued with overexpressed CENPE, these cells still only differentiated into type A colonies (Figure 5D; Supplementary Figure S8B; Supplementary Figure S9). Furthermore, the colony size of these cells increased to a similar levels as those in the control group (Supplementary Figure S8C). Taken together, overexpressed CENPE could not rescue the defective differentiation potential seen in RUNX1 shRNA-treated THP-1 cells.

Taken together, these results suggested that RUNX1 up-regulated CENPE to promote THP-1 cell growth by cell proliferation, but the differentiation potential involves other factors.

## Discussion

RUNX1 is pivotal to hematopoietic differentiation. Its dysregulation is often observed in leukemia. The role played by dysregulated wild-type RUNX1 expression plays in leukemia is still largely unknown. In normal hematopoietic development, RUNX1 is considered the primary transcription factor facilitating lineage differentiation (Ichikawa et al., 2013; Ichikawa et al., 2004). This role of RUNX1 is not as prevalent in leukemia as the literature suggests that RUNX1 mainly promotes leukemic cell growth (Keita et al., 2013). To differentiate between these two processes, we first examined transcriptome changes directly influenced by RUNX1 in THP-1 leukemia cells and CD34^+^ HSPCs. Among the top affected biological functions were those involving the cell cycle and proliferation (Figure 1B). When RUNX1 was knocked down in human THP-1 cells and CD34^+^ HSPCs, CD34^+^ cell counts were slightly altered, whereas in THP-1 cells, the cell counts were drastically reduced (Figure 2C). This depleted cell number phenotype in the RUNX1 KD THP-1 cell was similar to that observed in mouse MLL-AF9 and MLL-AF4 cells when treated with RUNX1 shRNA (Goyama et al., 2013; Wilkinson et al., 2013), This similarity indicated that the function of dysregulated RUNX1 in MLL fusion leukemia was likely conserved.

Two major players affecting changes in cell numbers are proliferation and apoptosis. We observed proliferation marker expression increased or reduced in THP-1 cells treated with overexpressed RUNX1 or RUNX1 shRNA, respectively (Figure 4A). This was consistent with the enriched biological functions involving the cell cycle and cell proliferation observed in the transcriptome profiles (Figure 1C), which suggested that RUNX1 could directly regulate cell proliferation. We also observed increased apoptosis marker expression when the THP-1 cell line was treated with RUNX1 shRNA, but no effects were observed when cells were treated with overexpressed RUNX1. Interestingly, in THP-1 cells, the anti-apoptotic maker BCL2 (Ruvolo et al., 2001) is up-regulated and RUNX1 has been reported to occupy its promoter region (Supplementary Figures S7A, S8D). This indicated that RUNX1 could also affect cell apoptosis, but the specific mechanisms involved require further research. Our results indicated that RUNX1 facilitates cell proliferation and provides leukemia cells with a growth advantage.

We further identified CENPE, a centromere-associated gene that accumulates in the G2 phase of the cell cycle (Abrieu et al., 2000; Yardimci et al., 2008), as a direct target gene of RUNX1. Over-expression of CENPE in THP-1 cells with RUNX1 knockdown rescued the cell number depletion and decreased cell proliferation, but did not increase apoptosis or impaired cell differentiation (Figures 5B–D). Furthermore, knockdown of CENPE expression in THP-1 cells resulted in both the reduction of cell numbers and cell proliferation, but showed no change in apoptosis (Supplementary Figures S7C,D). On the molecular level, compared with CD34^+^ cells, the expression of CENPE was higher in THP-1 cells (Figure 5A). RUNX1 occupied the promoter region of CENPE in the THP-1 cells but not in CD34^+^ cells (Figure 1D) and in the absence of RUNX1 expression in the THP-1 cells, CENPE expression was also abolished, while such changes were modest to undetectable in CD34^+^ cells (Figure 1E). These results indicate that the aberrantly expressed wild-type RUNX1 directly regulated CENPE expression to promote the growth advantage observed in THP-1 cells.

The current understanding of leukemia development supports a “2-hit” model of leukemogenesis. This model suggests that the pathogenesis of leukemia is caused by the double hits to the cell: one hit involves mutations of differentiation-associated genes such as RUNX1 and Etv1 (Grimwade et al., 2016; Zhao et al., 2014), which leads to aborted differentiation and accumulation of mutant cells; and the other hit involves mutations of proliferation-associated genes, such as Flt and Kit (Gilliland and Griffin, 2002), inducing the transformation of mutated cells into rapidly proliferating cells. Only once this dual dysfunction occurs, will leukemia manifest. RUNX1 mutations in leukemia are thought to play a role in differentiation dysregulation in this “2-hit” model (Conway O’Brien and Steven, 2014; Grimwade et al., 2016), albeit removing dysregulated wild-type RUNX1 in leukemia cells also causes a drastic reduction in cell numbers [Figure 2C, (Li et al., 2019; Sun et al., 2019)]. Our study suggests a potential mechanism whereby dysregulated wild-type RUNX1 promotes leukemic cell growth via altered regulation of cell proliferation modulated through the RUNX1-CENPE axis.

## Data Availability

The datasets presented in this study can be found in online repositories. The names of the repository/repositories and accession number(s) can be found in the article/Supplementary Material.
