# Corrigendum: RUNX1 Upregulates CENPE to Promote Leukemic Cell Proliferation

**DOI:** 10.3389/fmolb.2022.834509

**Published:** 2022-02-01

**Authors:** Shan Liu, Jianyu Yang, Guohuan Sun, Yawen Zhang, Cong Cheng, Jin Xu, Kuangyu Yen, Ting Lu

**Affiliations:** ^1^ School of Biology and Biological Engineering, Southern China University of Technology, Guangzhou, China; ^2^ State Key Laboratory of Experimental Hematology, National Clinical Research Center for Blood Diseases, Institute of Hematology and Blood Diseases Hospital, Chinese Academy of Medical Sciences and Peking Union Medical College, Tianjin, China; ^3^ Department of Developmental Biology, School of Basic Medical Sciences, Southern Medical University, Guangzhou, China; ^4^ Department of Cell Biology, Tianjin Medical University, Tianjin, China; ^5^ Division of Cell, Developmental and Integrative, School of Medicine, Southern China University of Technology, Guangzhou, China

**Keywords:** Runx1, leukemia cell, cell cycle, apoptosis, differentiation potential

During the revision experiments for this article, the authors repeated the experiments in THP-1 cells shown in Figures 3B, 5D to improve the quality of the images. As all five experimental conditions were done together (control, shRUNX1, CENPE OE, CENPE + shRUNX1, and RUNX1 OE), the control and shRUNX1 (labeled as sh2) panels in Figure 5D were repeated from panel Figure 3B. The authors have now removed the control and shRUNX1 panels in Figure 5D to avoid redundancy, and the legend has been updated to reflect that these data can be found in Figure 3B as well as in a new supplementary figure (Supplementary Figure S9) showing images for all five conditions in triplicate. The corrected Figure 5D and Supplementary Figure S9, corresponding figure legends, and text corrections appear below.

**FIGURE 5:**
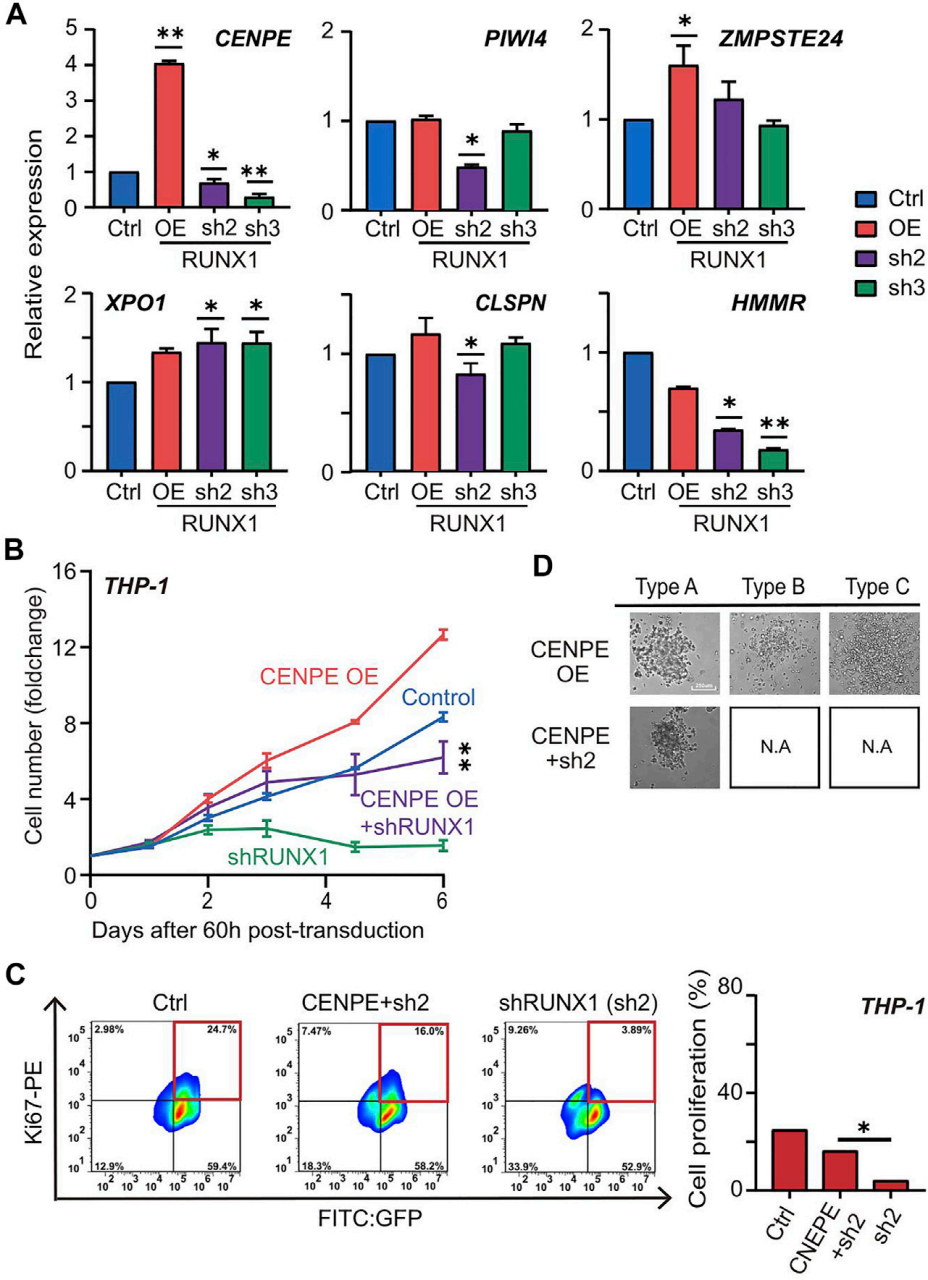
**(D)** The colony subtypes of THP-1 cells after transduction of CENPE (labeled as CENPE OE) and CENPE + shRUNX1 (labeled as CENPE + sh2) were observed under the microscope [control (labeled as Ctrl) and shRUNX1 (labeled as sh2) conditions are shown in Figure 3B, but not shown here to avoid redundancy]. Supplementary Figure S9 shows images for all conditions in triplicate.

Corrected Figure 5D and the corresponding figure legend appear below:

Supplementary Figure S9 and the corresponding figure legend appear below:

The text corrections appear below:

A correction has been made to the **Results** section, subsection “*RUNX1 Affects Leukemia Cell Growth and Differentiation*”, paragraph 5:

“For THP-1 cells, we observed three colony subtypes in THP-1 cells transduced with non-targeting lentivirus (Figure 3B; Supplementary Figure S9), which was similar to what was previously described for MLL-AF9 leukemia cells (Johnson et al., 2003). Notably, type A was the predominant colony subtype, while types B and C were less frequent (Figures 3C,D). Interestingly, we observed all three subtypes in the RUNX1 OE group; the RUNX1 KD group, on the other hand, formed only type A while types B and C almost completely disappeared (Figures 3B–D; Supplementary Figure S9).”

A correction has been made to the **Results** section, subsection “*RUNX1 Regulates CENPE to Promote Leukemia Cell Growth*”, paragraph 4:

“Using the CFU assay, we seeded 1000 THP-1 cells harboring RUNX1 shRNA and CENPE OE combined treatment in a 96-well plate for 15 days. As shown in Figure 3B, THP-1 cells transfected with no-targeting lentivirus (control) displayed three colony subtypes. Similarly, we observed three colony subtypes in THP-1 cells transfected with overexpressed CENPE (Figure 5D; Supplementary Figure S9). THP-1 cells that knocked down RUNX1 could only differentiate into type A but not type B or C cells (Figure 3B; Supplementary Figure S9). In addition, THP-1 with RUNX1 shRNA treatment displayed a reduced colony size (Figure 3B; Supplementary Figure S9). Interestingly, when rescued with overexpressed CENPE, these cells still only differentiated into type A colonies (Figure 5D; Supplementary Figure S8B; Supplementary Figure S9).”

In the original article, there was a mistake in Supplementary Figure S5C as published. Two images in Supplementary Figure S5C were mistakenly duplicated. The fluorescence images for 48 h Ctrl were repeated copies of the fluorescence images in 48 h sh1. Furthermore, the fluorescence images for 60 h Ctrl were repeated copies of the fluorescence images for THP-1 30 nM in Supplementary Figure S5A. The corrected Supplementary Figure S5C appears below.

The authors apologize for these errors and state that they do not change the scientific conclusions of the article in any way. The original article has been updated.

## Supplementary Material

Supplementary Figure S5 Virus concentration, cell doubling time, and transfection time point to optimize the transduction efficiency. **(A)** THP-1 cells treated with different concentration of RUNX1 shRNA from 5 to 30 nM. The transfection efficiency was observed under microscope using GFP. **(B)** The cell doubling time after transduction. Dotted boxes showed the normal doubling time of THP-1 cells. Sixty hours after transfection, cell doubling of each group recovered to the normal level. **(C)** FACS sorting time point to sort the successfully transducted cell at 48 and 60 h. Transfection efficiency was calculated as GFP ratio.

Supplementary Figure S9 Replicates for the CFU assay of THP-1 cells under different conditions. **(A)** Replicate 1, **(B)** replicate 2, and **(C)** replicate 3 for the colony subtype of THP-1 cells after transduction of non-targeting shRNA (labeled as Ctrl), shRUNX1 (labeled as sh2), CENPE (labeled as CENPE OE), CENPE + shRUNX1 (labeled as CENPE OE + sh2), and RUNX1 OE were observed under microscope. Replicate 1 was used for Figures 3B, 5D.

