# Supplementary material for: RUNX1 Upregulates CENPE to Promote Leukemic Cell Proliferation
Source: Front Mol Biosci. 2021 Aug 9;8:692880. doi: 10.3389/fmolb.2021.692880 (PMC8381024; doi:10.3389/fmolb.2021.692880)
Supplement: Supplementary file 1 [file DataSheet2.pdf]

Sup Fig 1. multiQC results on both ChIP-seq and RNA-seq. **(A)** Sequence counts for each sample in RNA-seq and ChIP-seq. **(B)** Sequence Quality Histograms of RNA-seq and ChIP-seq.

Sup Fig 2. Cell growth-related pathways contain predominantly up-regulated DEGs in THP-1 cells. **(A)** Left panel: qRT-PCR confirmed the relative RUNX1 mRNA expression between CD34+ and THP-1 cells. Right panel: Western blot confirmed the transducted result at protein level. THP-1 and CD34+ cells transducted with wildtype RUNX1, RUNX1 shRNA (sh1, sh2 and sh3) and non-targeting shRNA, each group immunoblot with anti RUNX1(ab23980). **(B)** Gene Ontology (GO) analysis of 5295 differentially expressed genes between THP-1 cells and CD34+ cells. Black font indicates the biological pathways that are related to cell growth; gray font indicates other biological pathways. **(C)** Go term analysis of THP-1 verse CD34+ cells differentially expressed genes. Red bar graph indicates the biological pathways of the up-regulated genes in THP-1 cells. Blue bar graph indicates the biological pathways of the down-regulated genes in THP-1 cells**. (D)** Go term analysis of THP-1 verse Monocyte differentially expressed genes. The gray bar graph indicates the biological pathways of the up-regulated genes in THP-1 cells.

Sup Fig 3. 167 out of the 1951 THP-1 potential target gene are considered as MLL-AF9 target genes. (A) Venn diagram showed the overlap of genes among THP-1 specific RUNX1 potential target genes and THP-1 MLL-AF9 target gene. (B). RUNX1 peaks located in the proximal vicinity of TSSs of genes (XPOI and CLSPN).

Sup Fig4. qPCR validation for Top 10 THP-1 specific RUNX1 potential target cell growth-related genes in THP-1 cells with or without RUNX1 shRNA. **(A)** qRT-PCR confirmed the relative mRNA expression among THP-1 treated with empty vector and RUNX1 shRNA. FANCD2 and ANLN, two cell growth-related genes that shown upregulation in THP-1 but contain no Runx1 binding on their promoter regions, are chosen as control. (one-way ANOVA, *p<0.05, **p<0.01, ***p<0.001; error bars, median ± SD).

Sup Fig 5. Virus concentration, cell doubling time, and transfection time point to optimize the transduction efficiency. (A) THP-1 cells treated with different concentration of RUNX1 shRNA from 5 to 30 nM. The transfection efficiency was observed under microscope using GFP. (B) The cell doubling time after transduction. Dotted boxes showed the normal doubling time of THP-1 cells. Sixty hours after transfection, cell doubling of each group recovered to the normal level. (C) FACS sorting time point to sort the successfully transducted cell at 48 and 60 h. Transfection efficiency was calculated as GFP ratio.

Sup Fig 6 RUNX1 knocked down affect PMA-induced differentiation. (A) RUNX1 Protein level of CD34+ and THP-1 cells treated with an empty plasmid, RUNX1 shRNA, and RUNX1. Upper panel: Western blot of RUNX1 from CD34+ and THP-1 cells treated with an empty plas3mid, RUNX1 shRNA, and RUNX1. GAPDH was

used as the internal control. Bottom panel: RUNX1/GAPDH quantification of Western blot data. (B) THP-1 cells underwent empty control, RUNX1 shRNA and wildtype RUNX1 were seeded at 200,000 cells per well in a 6-well plate. They were treated with 25 ng/ml PMA. Cells were imaged on 0 hours, 48 hours and 72 hours. The yellow arrows indicated a differentiated cell with adherent, flattened morphology. The quantification of cell morphological changed cells ratio among all groups (THP-1 cells underwent empty control, RUNX1 shRNA and wildtype RUNX1).

Sup Fig 7 Knocked down CENPE significantly reduce cell number and cell proliferation in THP-1 and RUNX1 OE THP-1 cells. **(A)** qRT-PCR confirmed the relative mRNA expression among THP-1 treated with wildtype RUNX1, RUNX1 shRNA (sh2 and sh3) and non-targeting shRNA. Top 10 THP-1 specific RUNX1 target genes involved in cell growth-related pathways were verified (table 1). **(B)** Growth curve of THP-1 and RUNX1 OE THP-1 cells that treated with CENPE shRNA. **(C)** and **(D)** FACS analysis of cell proliferation and cell apoptosis in THP-1 cells treated with Control, CENPE shRNA, RUNX1 and RUNX1+ CENPE shRNA. (one-way ANOVA, *$p<0.05$, **$p<0.01$, ***$p<0.001$; error bars, median ± SD).

Sup Fig 8. RUNX1 up-regulates CENPE to promote THP-1 cell growth by cell proliferation. **(A)** FACS analysis of cell apoptosis. Flow plots (left) and histograms (right) show the cell apoptosis ratio. **(B)** The whole colony number calculated at **(B)**, and colony size (lower) was respectively analyzed at **(C)**. **(D)** RUNX1 peaks located in the proximal vicinity of TSSs of BCL2 genes

Sup Fig 9. Replicates for the CFU assay of THP-1 cells under different conditions. (A) Replicate 1, (B) replicate 2, and (C) replicate 3 for the colony subtype of THP-1 cells after transduction of non-targeting shRNA (labeled as Ctrl), shRUNX1 (labeled as sh2), CENPE (labeled as
CENPE OE), CENPE + shRUNX1 (labeled as CENPE OE + sh2), and RUNX1 OE were observed under microscope. Replicate 1 was used for Figures 3B, 5D.
