# Supplementary figures and images for: RUNX1 Upregulates CENPE to Promote Leukemic Cell Proliferation
Source: Front Mol Biosci. 2021 Aug 9;8:692880. doi: 10.3389/fmolb.2021.692880 (PMC8381024; doi:10.3389/fmolb.2021.692880)

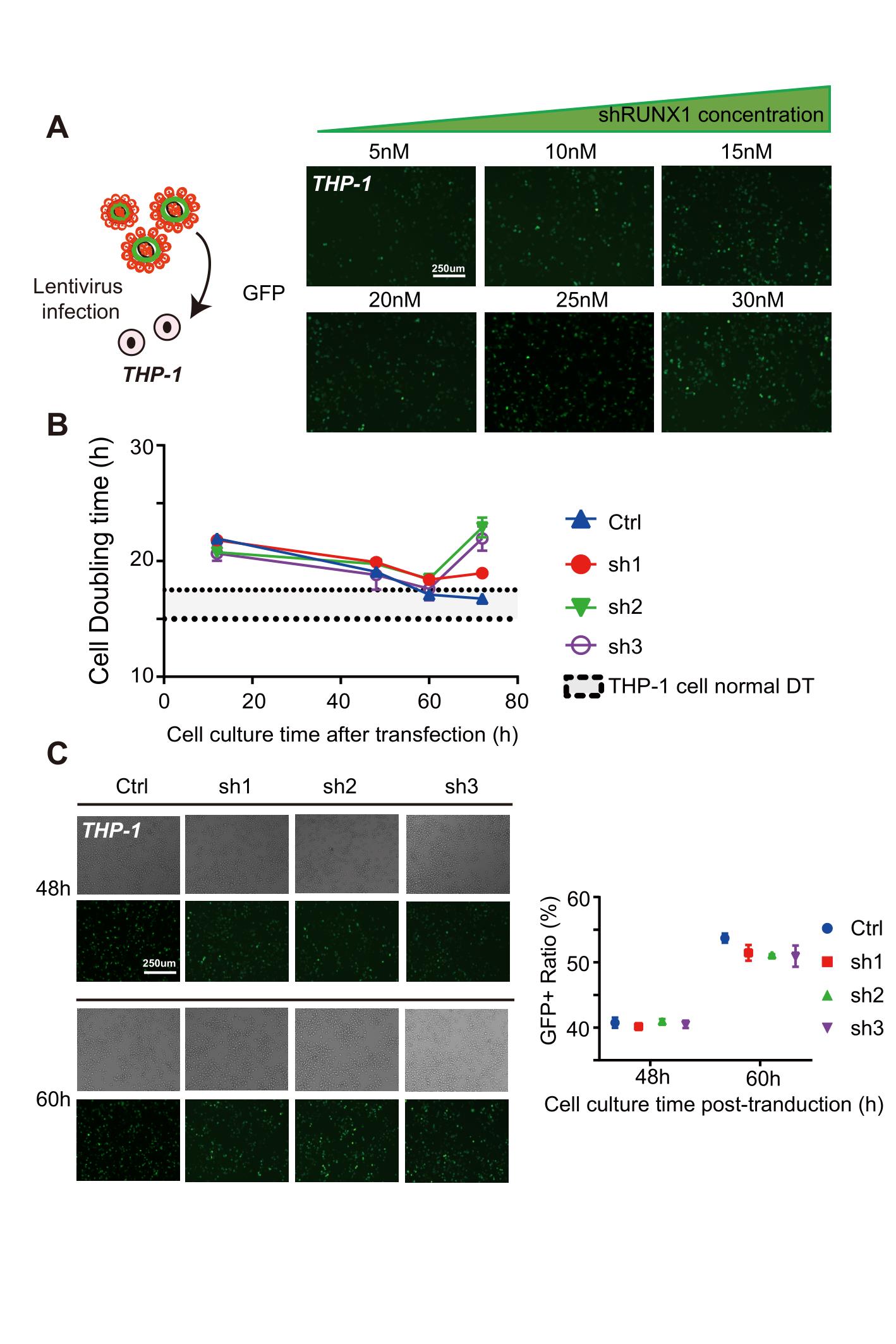

Supplement: Supplementary file 2 [file Image5.jpg]

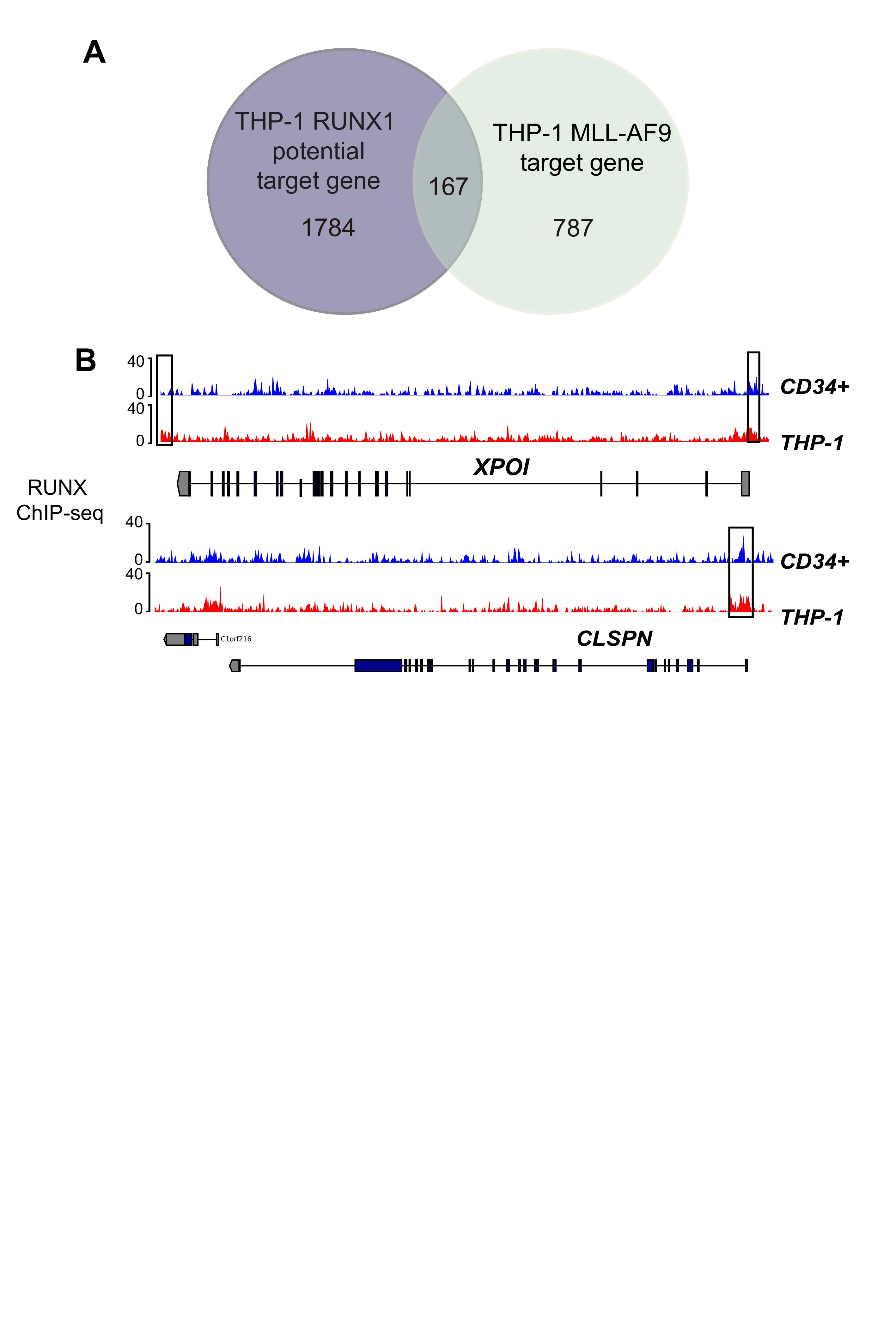

Supplement: Supplementary file 3 [file Image3.JPEG]

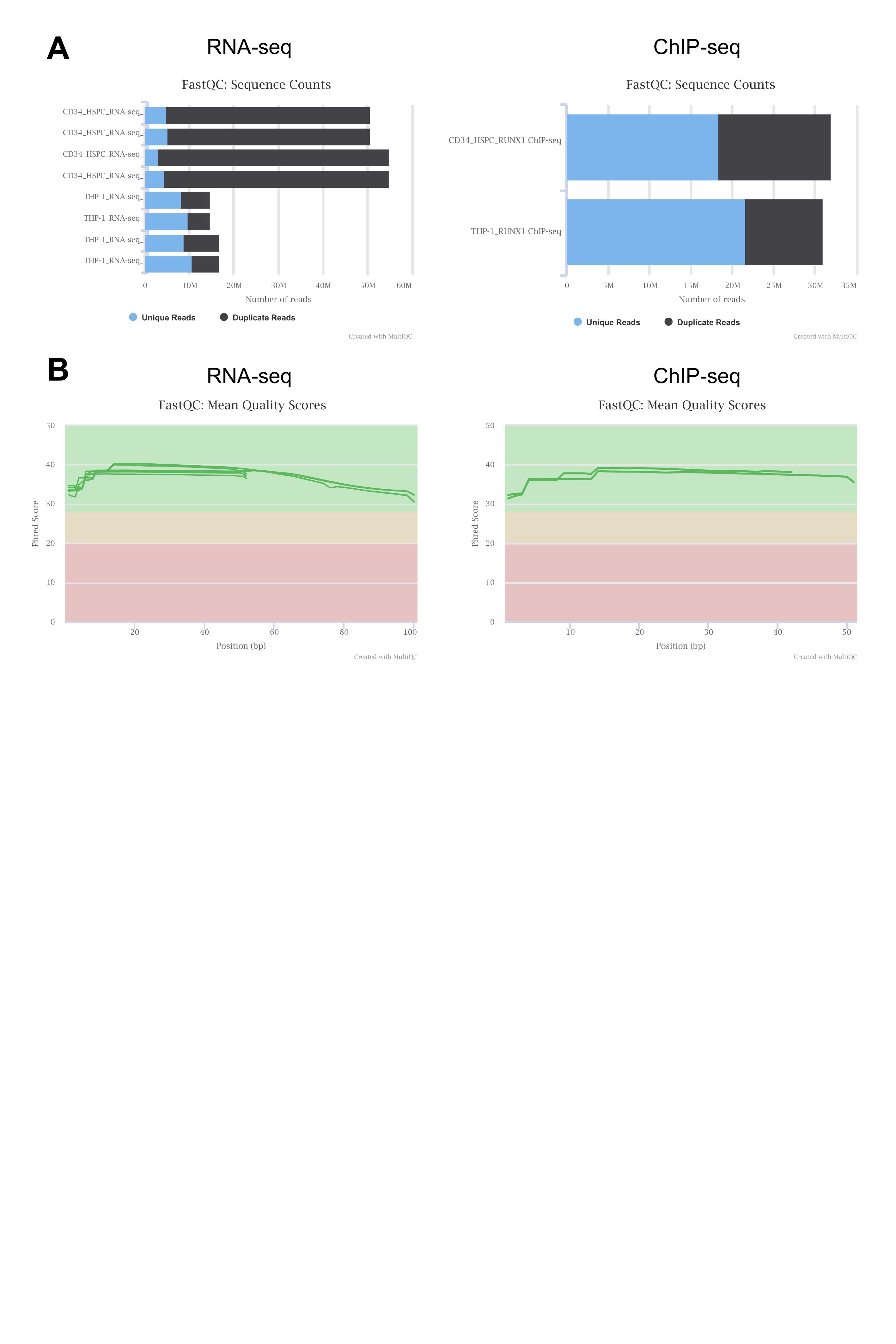

Supplement: Supplementary file 4 [file Image1.JPEG]

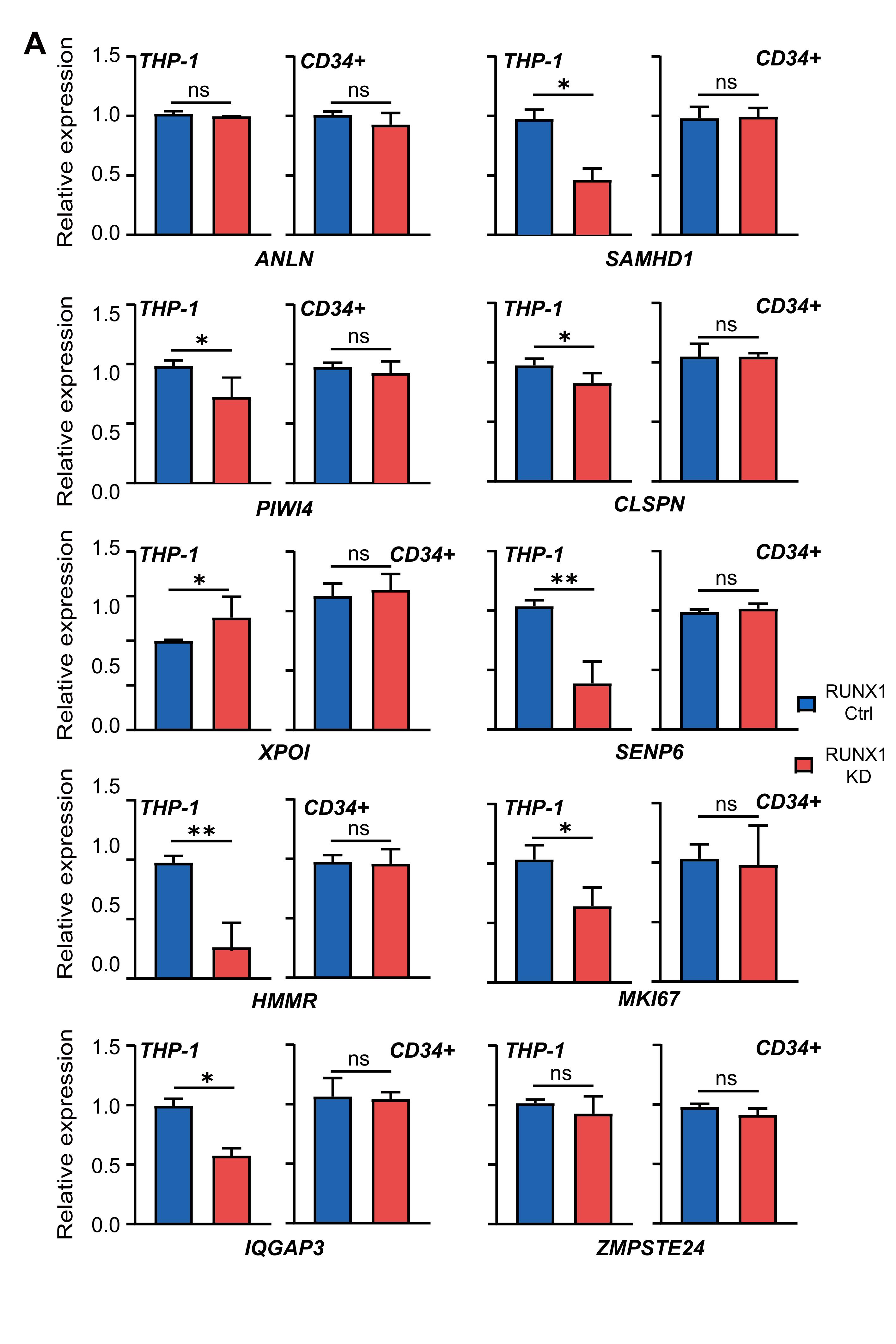

Supplement: Supplementary file 5 [file Image4.JPEG]

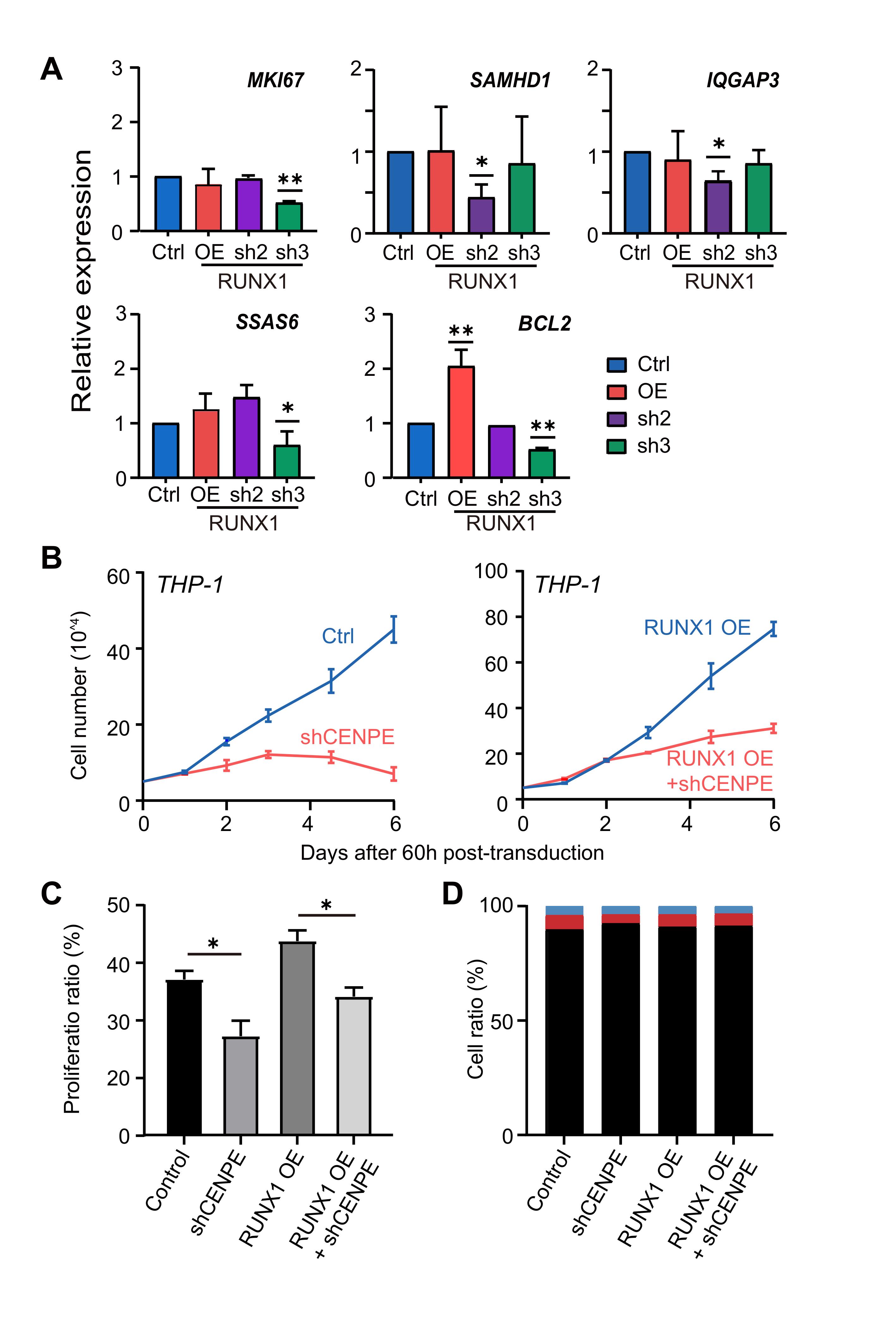

Supplement: Supplementary file 6 [file Image7.JPEG]

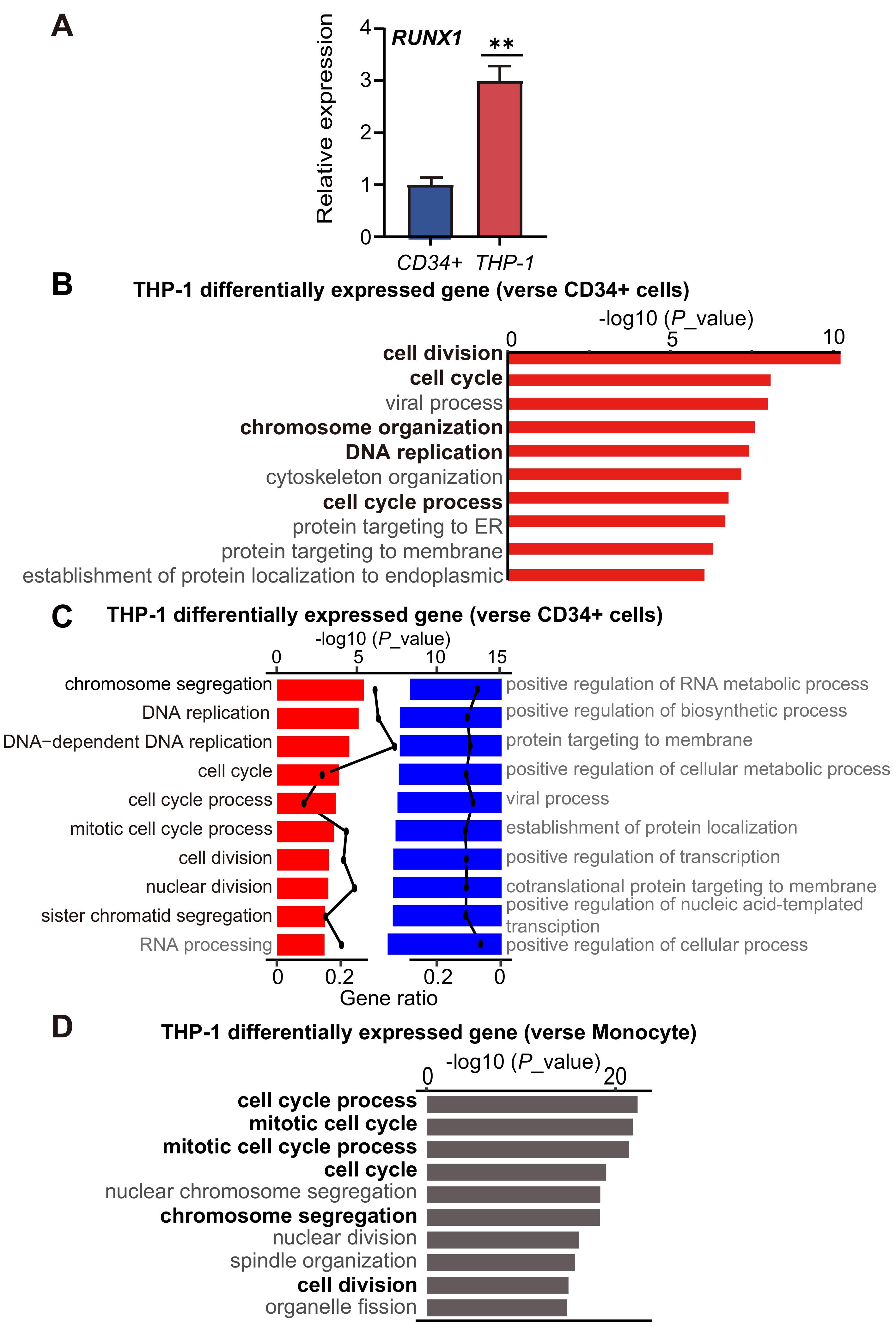

Supplement: Supplementary file 7 [file Image2.JPEG]

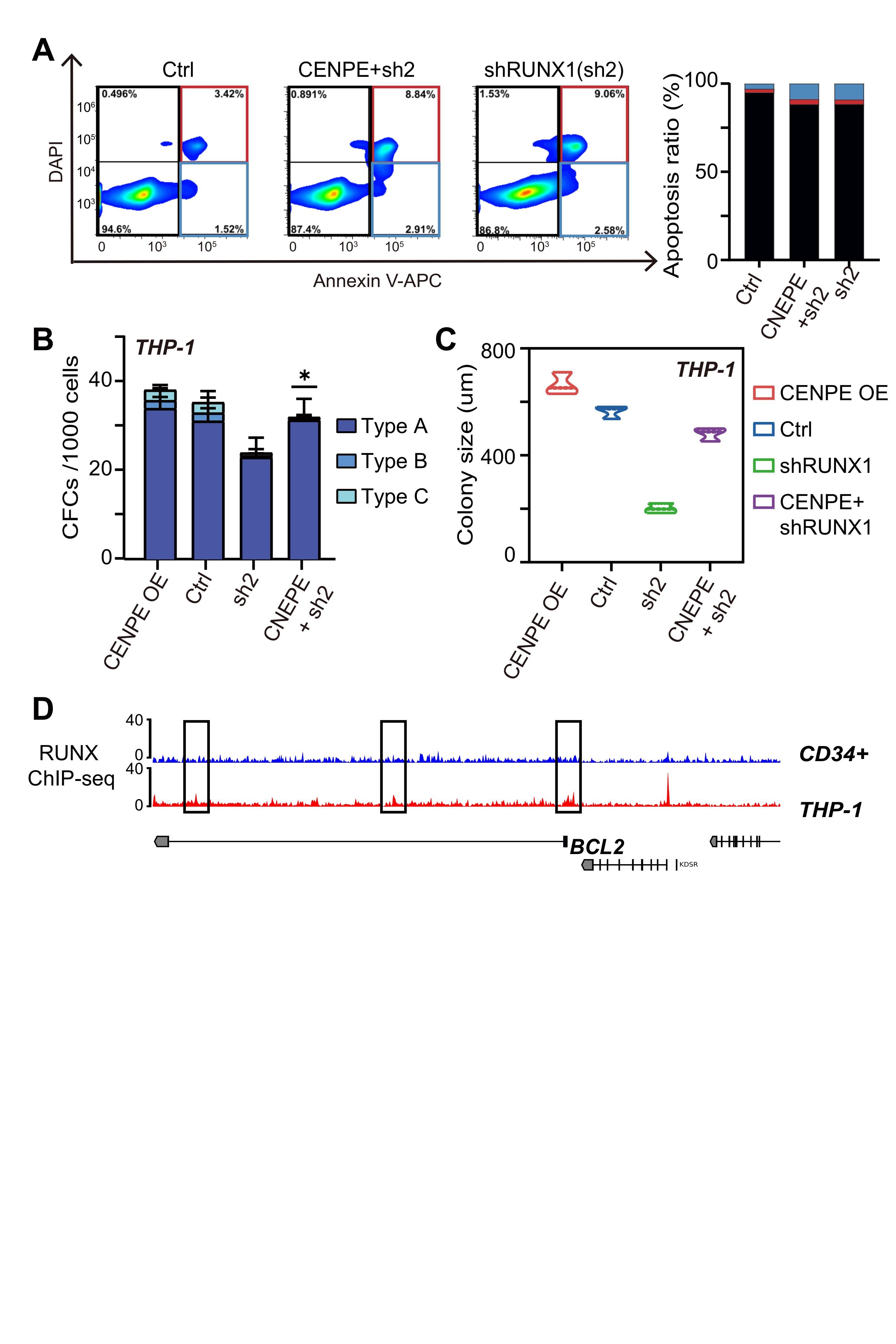

Supplement: Supplementary file 9 [file Image8.JPEG]

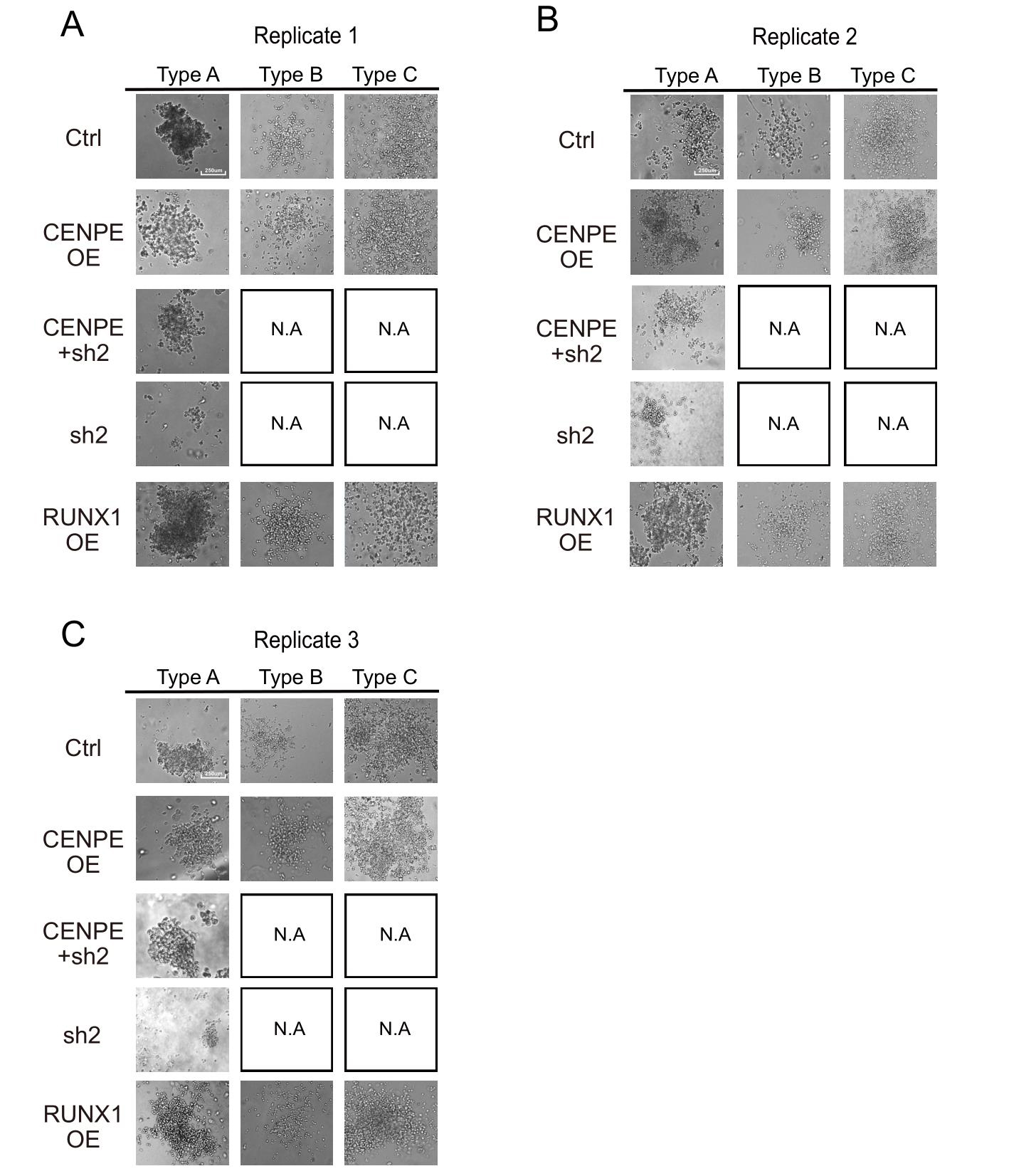

Supplement: Supplementary file 10 [file Image9.jpg]

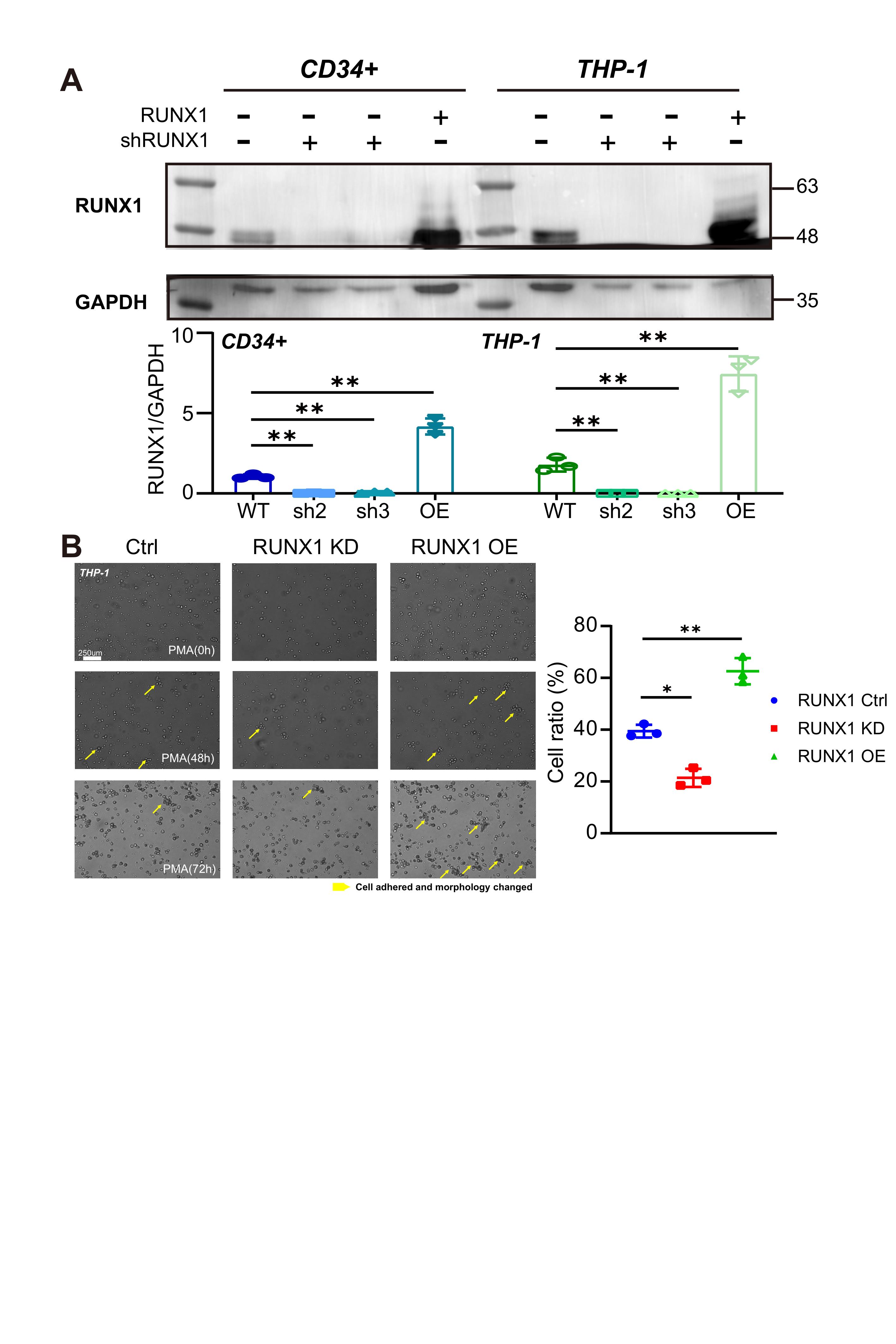

Supplement: Supplementary file 11 [file Image6.JPEG]
