# Supplementary material for: RUNX1 Upregulates CENPE to Promote Leukemic Cell Proliferation
Source: Front Mol Biosci. 2021 Aug 9;8:692880. doi: 10.3389/fmolb.2021.692880 (PMC8381024; doi:10.3389/fmolb.2021.692880)
Supplement: Supplementary file 8 [file DataSheet1.PDF]

Table 1 Genes in DNA replication and cell cycle biological process

Gene	GO process
CENPE	cell cycle / cell cycle process
ZMPSTE24	mitotic cell cycle process
CLSPN	G2/M transition of mitotic cell cycle
XPO1	cell cycle
SENP6	cell division
HMMR	mitotic cell cycle
PIWIL4	cell cycle /ncRNA metabolic process
SAMHD1	DNA replication
MKI67	RNA processing
IQGAP3	cell cycle / mitotic cell cycle phase transition

Table 2. qPCR primer

CENPE	Forward: GGAGAAAGATGACCTACAGAGGC/GCGATGGAAGAACAACTAGGTACC<br>Reverse: AGTTCCTCTTCAGTTTCCAGGTG/GTTGCTTGGGACTGTAAAAGCTGT
GAPDH	Forward: CTGACTTCAACAGCGACACCC<br>Reverse: CCCTGTTGCTCTAGCCAAAT
ZMPSTE24	Forward: CAAATTCACACCTCTGCCTGAGG<br>Reverse: GCATTGCTGTGGGAAGAGCGTT
CLSPN	Forward: CTCACTGCTAAACCAGCCCA<br>Reverse: ATGCTTCGCGTCAATCCTGA
XPO1	Forward: GAGCTCACTGGAAATTTCTGAAGA C<br>Reverse: CCAATCATGTACCCCACAGCTTC
HMMR	Forward: AGAAGTATTGAAAGGACCAGTATC/CAGGTCACCCAAAGGAGTCTCG<br>Reverse: TTCAAATCTTTATCATTCTTTTGA/CCACTTGATCTGAAGCACAACTAA
PIWIL4	Forward: AATGCTCGCTTTGAACTAGAGAC<br>Reverse: ATTTTGGGGTAGTCCACATTAAATC
SAMHD1	Forward: GCTCTGCAAATTTCTCTGGCAG<br>Reverse: CTGACTTCAACAGCGACACCC
MKI67	Forward: GCTCTGCAAATTTCTCTGGCAG<br>Reverse: CTGACTTCAACAGCGACACCC
IQGAP3	Forward: GCTCTGCAAATTTCTCTGGCAG<br>Reverse: CTGACTTCAACAGCGACACCC
