# Supplementary figures and images for: Corrigendum: RUNX1 Upregulates CENPE to Promote Leukemic Cell Proliferation
Source: Front Mol Biosci. 2022 Feb 1;9:834509. doi: 10.3389/fmolb.2022.834509 (PMC8889725; doi:10.3389/fmolb.2022.834509)

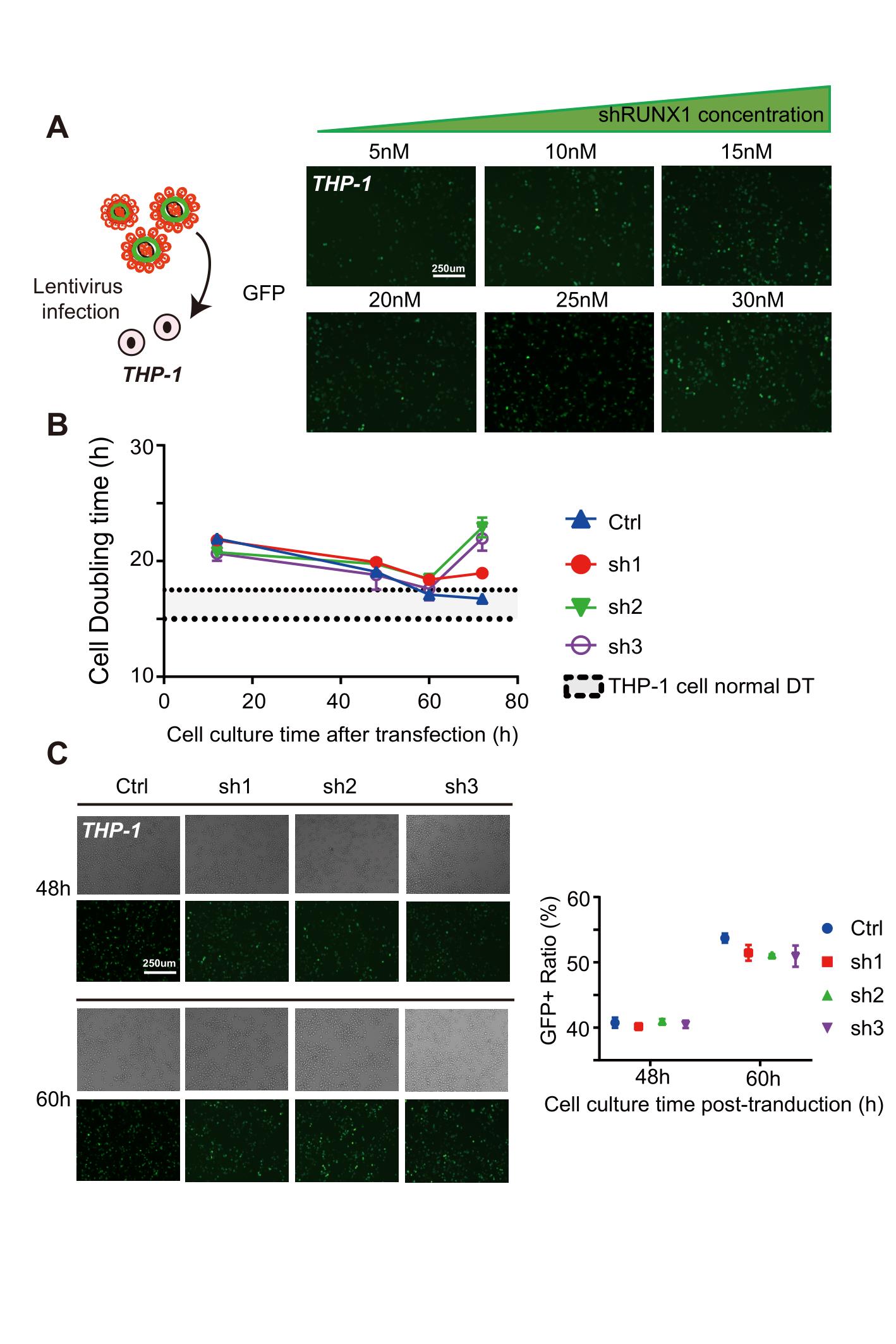

Supplement: Supplementary file 1 [file Image5.jpg]

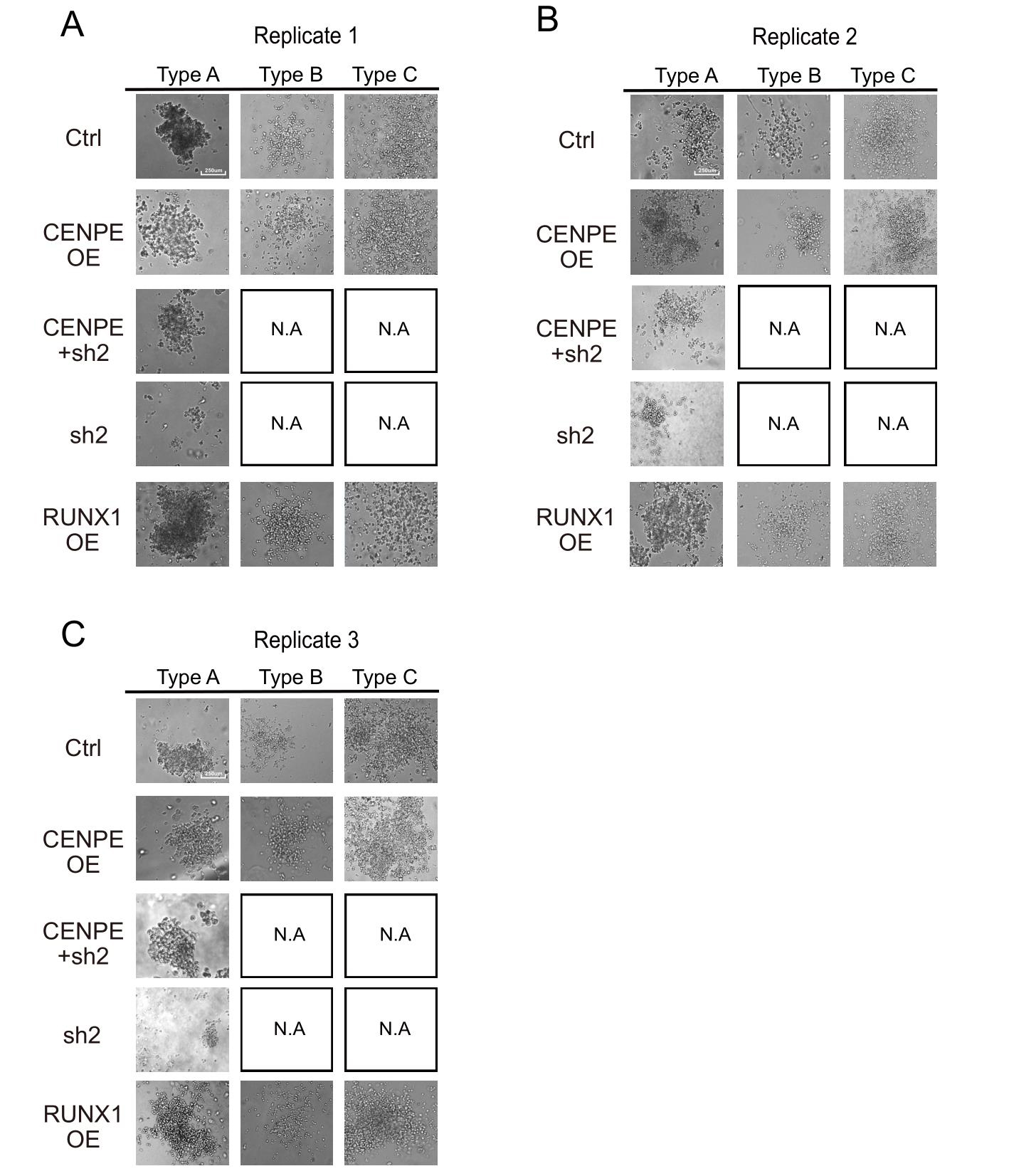

Supplement: Supplementary file 2 [file Image9.jpg]
